# A BRN2:MYC transcriptional axis regulates interconversion between therapy-resistant and tumorigenic phenotypes in melanoma

**DOI:** 10.1016/j.celrep.2025.116675

**Published:** 2025-12-15

**Authors:** Yuntian Zhang, Marcus A. Urquijo, Rebecca G. Zitnay, Kayla Marks, Rachel L. Belote, Maike M.K. Hansen, Montana Ferita, Hannah M. Neuendorf, Tong Liu, Eric A. Smith, Elnaz Mirzaei Mehrabad, Miroslav Hejna, Tarek E. Moustafa, Devin Lange, Min Hu, Fatemeh Vand-Rajabpour, Anne Done, Carly A. Becker, Matthew Lieberman, Matthew Chang, Brian K. Lohman, Chris J. Stubben, Melissa Q. Reeves, Xiaoyang Zhang, Leor S. Weinberger, Matthew W. VanBrocklin, Dekker C. Deacon, Douglas Grossman, Benjamin T. Spike, Alexander Lex, Glen M. Boyle, Rajan Kulkarni, Thomas A. Zangle, Robert L. Judson-Torres

**Affiliations:** 1 Helen Diller Family Comprehensive Cancer Center, University of California, San Francisco, San Francisco, CA 94158, USA; 2 Huntsman Cancer Institute, University of Utah, Salt Lake City, UT 84112, USA; 3 Department of Oncological Sciences, University of Utah School of Medicine, Salt Lake City, UT 84103, USA; 4 Department of Molecular Genetics, The Ohio State University College of Arts and Sciences, Columbus, OH 43210, USA; 5 Department of Chemical Engineering, University of Utah, Salt Lake City, UT 84103, USA; 6 Department of Dermatology, University of Utah School of Medicine, Salt Lake City, UT 84132, USA; 7 Radboud University, Institute for Molecules and Materials, Heyendaalseweg 135, 6525 AJ Nijmegen, the Netherlands; 8 Department of Mathematics, University of Utah, Salt Lake City, UT, USA; 9 Cancer Drug Mechanisms Group, Cancer Research Department, QIMR Berghofer, Brisbane, QLD 4006, Australia; 10 School of Biomedical Sciences, Faculty of Health, Queensland University of Technology, Brisbane, QLD 4059, Australia; 11 Department of Pathology, University of Utah, Salt Lake City, UT 84132, USA; 12 Department of Physics, University of Illinois at Urbana-Champaign, Urbana, IL 61801, USA; 13 Carl R. Woese Institute for Genomic Biology, University of Illinois at Urbana-Champaign, Urbana, IL 61801, USA; 14 SCI Institute and Kahlert School of Computing, University of Utah, Salt Lake City, UT 84112, USA; 15 Department of Medical Genetics, School of Medicine, Tehran University of Medical Sciences, Tehran, Iran; 16 Department of Dermatology, Oregon Health and Science University, Portland, OR 97239, USA; 17 Departments of Biomedical Engineering and Oncological Sciences, Oregon Health and Science University, Portland, OR 97239, USA; 18 Cancer Early Detection Advanced Research Center, Knight Cancer Institute, Oregon Health and Science University, Portland, OR 97239, USA; 19 Operative Care Division, VA Portland Health Care System, Portland, OR 97239, USA; 20 Gladstone Center for Cell Circuitry, Gladstone Institutes, San Francisco, CA 94158, USA; 21 Departments of Pharmaceutical Chemistry and Biochemistry and Biophysics, University of California, San Francisco, San Francisco, CA 94158, USA; 22 Department of Surgery, University of Utah School of Medicine, Salt Lake City, UT 84132, USA; 23 School of Biomedical Science, Faculty of Health, Medicine and Behavioral Sciences, University of Queensland, Brisbane, QLD 4072, Australia; 24 These authors contributed equally; 25 Lead contact

## Abstract

Metastatic spread and therapeutic resistance are the principal causes of cancer mortality. For melanoma, these processes rely on the capacity of cells to switch between transcriptional states. Although targeting transcriptional states pharmacologically is promising, the mechanisms by which melanoma cells switch between states—and how these processes differ from melanocytes—remain poorly understood. Here, we isolate distinct melanoma states with unique phenotypes: a MYC-driven state, essential for tumor initiation yet sensitive to BRAF inhibition, and a dedifferentiated, invasive BRN2-high state enriched in therapy-resistant cells but not directly tumorigenic. Transitions between phenotypes occur through intermediate, more differentiated states. Unexpectedly, the BRN2-high state is also present in melanocytes, whereas the MYC state is exclusive to melanoma. Melanoma cells also exhibit an increased frequency of transitions across states. These findings highlight that accelerated phenotypic switching, rather than mere state diversity, is a defining feature of melanoma progression.

## In brief

Zhang et al. identify transcriptional states in melanoma, defining an MYC-driven tumor-initiating state and a BRN2-high invasive, therapy-resistant state bridged by MITF-high intermediates. While melanocytes share most states, melanoma uniquely acquires the MYC state and transitions more frequently, revealing plasticity as a therapeutic vulnerability.

## Graphical Abstract

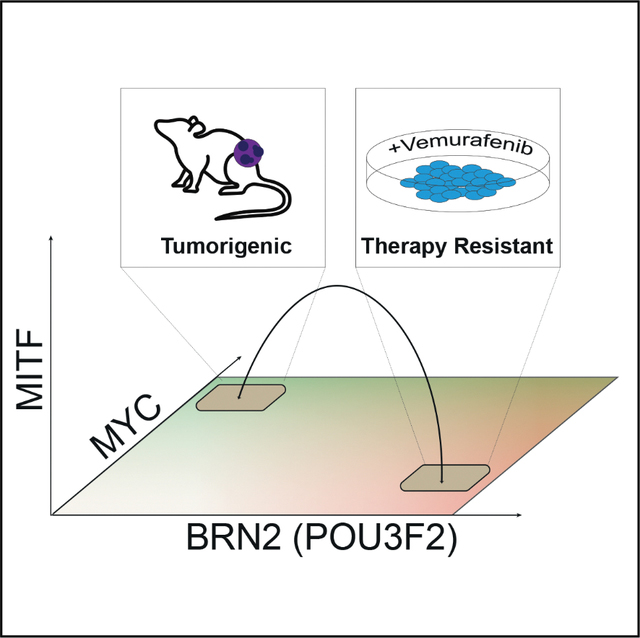

## INTRODUCTION

The skin cancer melanoma is often fatal in its advanced stages after the disease has metastasized to other organs.^1^ Despite recent progress in pharmacotherapy for advanced melanoma, around half of the patients still succumb to the disease, commonly due to acquired resistance to therapeutic agents. The processes of both metastatic dissemination and the development of therapeutic resistance are enabled by the ability of melanoma cells to reversibly interconvert between distinct transcriptional states.^2–5^ This adaptability facilitates their survival and proliferation under diverse environmental pressures. Thus, one promising avenue for discovering new therapeutic approaches lies in identifying agents that selectively eliminate or block transitions to notably aggressive transcriptional states.^4–11^ Such approaches hinge on the ability to consistently link some states with aggressive phenotypes, and other states with non-aggressive or benign attributes.

Attempts to prospectively isolate distinct transcriptional states have been unreliable, preventing the direct functional comparison of live cells.^12–20^ For example, pioneering studies on phenotype switching in melanoma introduced a dichotomy between invasive and proliferative cell states, distinguished by the expression of the BRN2 and MITF transcription factors, respectively.^21–25^ Although conducted on bulk cell populations, one theoretical concept that emerged from these studies was that of bidirectional phenotype switching of a single cell. This concept was investigated by Pinner and colleagues with a B16 mouse melanoma cell line engineered with a fluorescent reporter of *BRN2* promoter activity. *BRN2*-expressing cells were enriched among the invasive and circulating populations but were rare in both primary and metastatic tumors.^26^ These observed patterns, while consistent with the phenotype-switching model, do not preclude the alternative hypothesis of phenotype selection at different stages of metastatic dissemination. Indeed, Campbell and colleagues recently used a zebrafish melanoma model to demonstrate that both invasive and proliferative states stably coexist in heterotypic clusters during metastatic dissemination, with the invasive state required for cluster intravasation.^27^ These observations suggest a phenotype selection and enrichment model where distinct phenotypes remain stable within small heterogeneous cell populations and divide the responsibilities of invasion/intravasation and proliferation/extravasation. These two models, phenotype switching and phenotype selection and enrichment, are challenging to distinguish experimentally because both can yield similar patterns in the distribution of cell phenotypes at different stages of tumor progression. Both models predict that proliferative cells will dominate in primary and metastatic tumors, while invasive cells will be more prevalent in circulation. Thus, observed shifts in phenotypic prevalence at different stages are difficult to attribute to a specific mechanism (e.g., phenotypic switching versus clonal selection) when relying solely on sampling at different stages, rather than tracking individual cells over time.

Other reports similarly challenge the dichotomous invasion/proliferation phenotype-switching model. Melanoma cells are capable of simultaneous invasion and division.^28^ Conditions that induce the invasive phenotype or oppose the proliferative *MITF* program do not necessitate increased *BRN2* expression.^29–31^ In addition, dependent on the context, *MITF* and *BRN2* can also present reciprocal activation or no relationship at all.^21,22,24,29,31–34^ Moreover, recent single-cell RNA sequencing studies have expanded the landscape of melanoma cell phenotypes within clonal populations,^2–4,35–37^ yet these analyses have not reported the *BRN2* high phenotype identified by earlier studies. These observations are collectively inconsistent with the existence of a compulsory mutually exclusive *MITF*/*BRN2* program switch. Regardless of its relationship to *MITF*, *BRN2* expression oscillates between distinct stages of metastasis *in vivo*, and the gene itself can serve to either promote or suppress melanoma progression.^26,38,39^ Thus, the dynamic expression of *BRN2*, rather than its constant presence or absence, may be critical for melanoma progression.

Rather than a binary *MITF*/*BRN2* program, single-cell approaches have uncovered a spectrum of phenotypic states, including invasive, pigmented, neural crest-like, and undifferentiated phenotypes, among others.^40^ How melanoma cells might transition between these states has been inferred through computational prediction (such as pseudotime analyses) and experimental techniques that employ cell selection (such as pharmacologic or genetic manipulation); yet neither approach can confirm whether the induction of a new phenotype represents true phenotype-switching or phenotype selection and enrichment.^4,5,41^ Another area of uncertainty is the degree of similarity or difference between the transcriptional states identified in malignant cells and those of their putative healthy counterparts, the epidermal melanocytes in human skin. We and others have reported the pharmacologic induction of phenotype switching in healthy human epidermal melanocytes, raising pivotal questions about whether phenotype switching is fundamentally altered in melanoma cells or an inherent property of the melanocytic lineage.^33,42,43^

Here, we employed a transgenic fluorescent reporter of human *BRN2* promoter activity and directly observed bidirectional switching between an invasive phenotype characterized by high BRN2 expression, required for therapeutic resistance and a tumorigenic phenotype characterized by high MYC activity. The transition between phenotypes occurred through a third transcriptional state characterized by the expression of programs associated with differentiation. Surprisingly, we find that primary adult melanocytes in healthy skin can also express the dedifferentiation program, but that spontaneous fluctuations of state, as well as the *MYC* program, appear to be exclusive to melanoma cells. This work unifies the foundational observations of phenotype switching with recent single-cell transcriptomic insights, provides direct evidence of interconversion in human cells, and identifies the rate of state change as a fundamental distinction between healthy and malignant cells.

## RESULTS

### Dynamic BRN2 expression results in phenotypic heterogeneity in clonal populations

**Figure 1.**
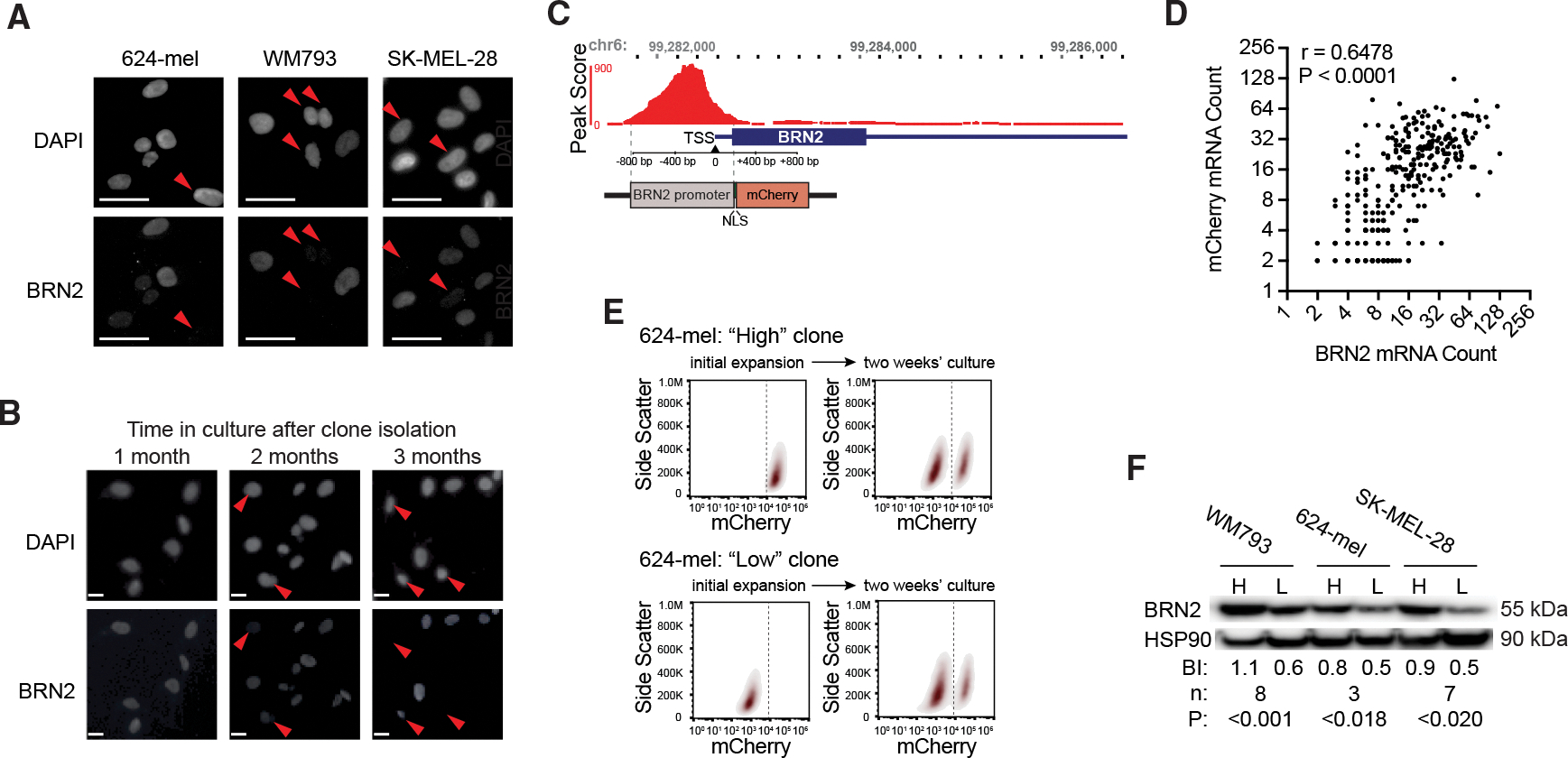
Gradual establishment of BRN2 expression equilibrium in human melanoma cells (A) Images of BRN2 immunofluorescence (IF) staining of the indicated cell lines. Red arrowheads indicate cells identified by DAPI staining (top row) that exhibit low BRN2 expression (bottom row). Scale bars, 20 μm. (B) Images of BRN2 IF in clonal expansion of 624-mel cells after 1, 2, and 3 months in culture. Scale bars, 20 μm. Quantification of fluorescence intensity is presented in Figures S1A and S1B. (C) ATAC-seq peak scores from the BRN2 locus (top) and schematic of reporter construct (bottom). TSS, transcriptional start site. NLS, nuclear localization signal. (D) Single-molecule fluorescent *in situ* hybridization BRN2 and mCherry transcript counts from individual 624-mel cells. r, Pearson’s correlation coefficient. P, two-tailed *p* value. (E) Flow analysis of clonally expanded and serially cultured 624-mel cells with the BRN2 reporter. Additional cell lines are presented in Figure S1C. (F) Western blot of BRN2 and HSP90 loading control in reporter high (H) and reporter low (L) cells from the indicated cell lines. BI, average blot intensity. n, number of independent lysates. P, two-tailed *t* test *p* value.

To determine the levels of BRN2 expression across cell populations, we employed immunofluorescence (IF) on three human melanoma cell lines. Each line exhibited heterogeneous BRN2 expression, with subsets of cells showing high or undetectable levels (Figure 1A). To determine whether this heterogeneity reflected irreversible genetic change or phenotype switching, we generated clonal populations of 624-mel cells and monitored BRN2 protein expression over time. BRN2 expression was uniform in short-term culture and became more heterogeneous over time (Figures 1B, S1A, and S1B). Regardless of the initial profile, the broad distribution of BRN2 expression characteristic of the parental population was re-established over an extended culture, suggesting phenotype switching but not confirming bidirectional interconversion.

To directly track phenotypic switching, we generated a fluorescent reporter construct for the promoter activity of *BRN2*. assay for transposase-accessible chromatin (ATAC)-seq in the 624-mel cell line identified the proximal active promoter region spanning from ~817 base pairs (bp) upstream of the transcriptional start site to ~195 bp into the untranslated transcript, consistent with prior reports in mouse (Figure 1C).^26^ We cloned this region into a selectable lentiviral mammalian expression construct, adjacent to a nuclear localization signal tagged mCherry, and transduced the 624-mel, WM793, and SK-MEL-28 human melanoma cell lines. Single-molecule fluorescence *in situ* hybridization confirmed a correlation between the expression of the mCherry and endogenous *BRN2* transcripts (Figure 1D). Initially, uniform clonal populations developed bimodal distributions of mCherry expression over two weeks in culture, such that mCherry low populations (“low^BRN2^”) begot mCherry high populations (“high^BRN2^”) and vice versa (Figures 1E and S1C), consistent with the redistributions of BRN2 expression observed via IF. To validate reporter fidelity, we used fluorescence-activated cell sorting (FACS) to isolate mCherry low and high cells from each population. The low^BRN2^ cells expressed 2- to 3-fold less BRN2 protein and mRNA as compared to high^BRN2^ cells (Figures 1F and S1D). We conclude that melanoma cell lines exhibit heterogeneous BRN2 expression that re-establishes after isolation and clonal outgrowth, and that mCherry expression from the reporter faithfully reports endogenous BRN2 levels.

### Distinct aggressive phenotypes are linked to high and low BRN2 expression

**Figure 2.**
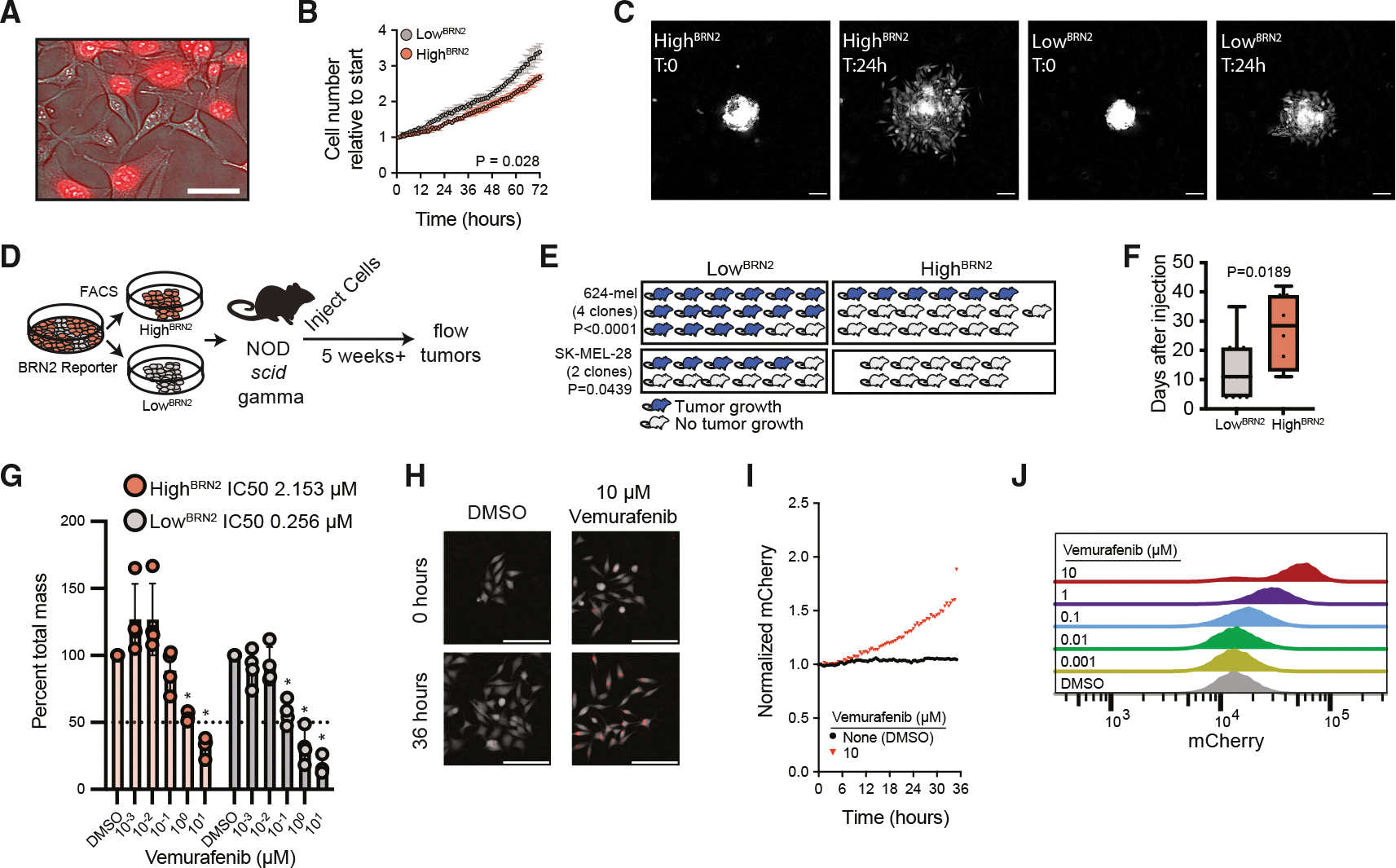
BRN2-mCherry high and low cells exhibit distinct phenotypes (A) Merged phase-contrast and fluorescent image of clonal 624-mel population expressing the BRN2-mCherry reporter. The quantification of morphological features of mCherry high (high^BRN2^) and mCherry low (low^BRN2^) cells is presented in Figure S2A. A heterogeneous expression pattern is evident. Scale bars, 50 μm. (B) Cell number (normalized to the start of the experiment) over time of high^BRN2^ and low^BRN2^ cells assessed with quantitative phase imaging. The population mean, standard deviation and *p* value from two-tailed *t* test (*n* = 3, independent experiments). (C) Representative images of spheroid assay from the starting time point (T = 0) and the final time point (T = 24 h). Scale bars, 100 μm. The mean and standard deviation across biological replicates are presented in Figure S2C. (D) Schematic of graft efficiency assay. Clonal 624-mel or SK-MEL-28 cultures were either depleted (red) or enriched (gray) for low^BRN2^ cells and implanted into NOD scid gamma mice. (E) Percent of mice with successful grafts after 5 weeks. P, two-tailed Fisher’s exact test *p* value. (F) First day after engraftment that a palpable tumor was detected. Box and whisker plot with the center line representing the median, boxes representing the interquartile range, and whiskers representing the minimum and maximum values, *p* value from two-tailed Fisher’s exact test. *N* (624MEL) = 37 mice and *n* (SK-MEL-28) = 22 mice. (G) Percent total cell mass after 48 h of exposure to the indicated concentrations of vemurafenib relative to the DMSO condition. The asterisk indicates the means are significantly different from the DMSO condition (mean mass, standard deviation, *p* < 0.0005, unpaired *t* test) (*n* = 3, independent experiments). (H) Representative image of low^BRN2^ cells treated with DMSO or 10 μM vemurafenib. Scale bars, 200 μm. (I) Normalized mean integrated mCherry intensity over time of FACS-enriched low^BRN2^ 624-mel cells exposed to the indicated concentrations of vemurafenib. (J) Flow analysis of mCherry expression from 624-mel reporter cells after 48 h of treatment with the indicated concentrations of vemurafenib.

While culturing, we noticed that low^BRN2^ cells were smaller and more uniform in appearance compared to the high^BRN2^ cells (Figure 2A). We isolated the populations and performed digital holographic cytometric feature classification—a method that compares aggregate morphological features of the cells.^44–46^ In all lines, low^BRN2^ cells were morphologically distinct from high^BRN2^ cells (*p* < 0.0001) (Figure S2A). We hypothesized that the morphological distinction might indicate differences in phenotypes.

We first explored the phenotypic differences between the lines in two-dimensional culture. Low^BRN2^ cells divided more rapidly than high^BRN2^ cells (doubling times of 19.5 h versus 25.5 h, respectively, *p* value = 0.0028) (Figure 2B). Neither line presented greater random motility in two-dimensional culture in the absence of extracellular matrix (Figure S2B). However, high^BRN2^ cells exhibited significantly more outgrowth from spheroids grown on a two-dimensional fibronectin-coated substrate (Figures 2C and S2C), consistent with the prior associations of BRN2 expression and an invasive phenotype.^23–25^

We next investigated whether low^BRN2^ or high^BRN2^ phenotypes impacted melanoma growth or metastatic behavior. One phenotype associated with aggressive disease is the ability to initiate tumorigenesis or metastasis upon engraftment into mice.^47,48^ We tested tumorigenicity using a graft efficiency assay. One million FACS-enriched low^BRN2^ or high^BRN2^ 624-mel (4 independent clones) or SK-MEL-28 (3 independent clones) cells were implanted subcutaneously into male and female non-obese diabetic (NOD) *scid* gamma mice (Figure 2D). At 5 weeks, low^BRN2^ cells were significantly more tumorigenic than high^BRN2^ cells (Figure 2E), forming tumors in 70% (21/30) of mice versus high^BRN2^, forming in 20.6% (6/29, *p* = 0.00002). Additionally, tumors from low^BRN2^-implanted mice formed more quickly than those implanted with high^BRN2^ cells (Figure 2F), though once formed, growth rates were comparable (Figure S2D). Mouse sex did not affect tumor onset or growth rate (Figures S2E and S2F). Primary tumors were dissociated, and human tumor cells were analyzed by flow cytometry. Independent of the reporter status of the injected cells, all tumors contained both mCherry low and mCherry high populations (Figure S2G). A greater proportion of injected high^BRN2^ cells converted to the low state, consistent with low^BRN2^ cells being more tumorigenic. In some cases, distant metastases were also detected and analyzed, revealing a similar predominance of low^BRN2^ cells within metastatic lesions. Collectively, these findings indicate that low^BRN2^ cells possess substantially greater tumor-initiating capacity than high^BRN2^ cells.

To monitor metastatic dissemination, a reporter 624-mel clone was transduced with a constitutively active luciferase construct. Two million FACS-enriched low^BRN2^ or high^BRN2^ cells were isolated and implanted subcutaneously into NOD *scid* gamma mice. Tumor growth was monitored over a five-week period, after which surviving mice were sacrificed and the brain, lungs, and liver were imaged (Figures S2H–S2J). Consistent with previous results, low^BRN2^ cells exhibited greater tumorigenicity than high^BRN2^cells (Figure S2K). All mice that developed primary tumors displayed distant metastases, as evidenced by luciferase activity (Figure S2L). Notably, none of the high^BRN2^-implanted mice without a palpable tumor exhibited detectable luciferase activity at either the primary injection site or at distant organs. These findings suggest that, in the absence of the tumorigenic low^BRN2^ state, insufficient viable cells persist to support metastatic seeding.

Resistance to therapeutic intervention is another feature of aggressive disease. Each of the three lines selected for this study harbors the *BRAF*^*V600E*^ oncogenic driver, identified in approximately half of all melanomas.^49^ Small molecules such as vemurafenib, dabrafenib, and encorafenib selectively target the BRAF^V600E^ mutation and serve as the primary treatment options for BRAF^V600E^-positive melanomas. Although these agents initially elicit clinical response, resistance develops widely in most patients.^50^ We FACS-isolated low^BRN2^ and high^BRN2^ 624-mel cells and conducted vemurafenib response studies. We first used ptychography coupled with fluorescence microscopy to track the accumulation of cell mass over time—a sensitive readout of changes for drug response.^51,52^ Exposure to vemurafenib inhibited cell mass accumulation in both populations with the high^BRN2^ populations exhibiting an IC50 ~10-fold higher than that of low^BRN2^ cells (2.15 μM, 95% confidence interval [CI]: 0.73–6.37 compared to 0.26 μM, 95% CI: 0.15–0.45, *p* < 0.0005, unpaired *t* test), indicative of greater tolerance to the inhibitor (Figures 2G and S2M). Exposure to vemurafenib also increased mCherry expression (Figures 2H and 2I), and flow cytometry analysis showed surviving cells uniformly upregulated mCherry, depleting low^BRN2^ cells (Figure 2J). Together with graft assays, these results show that BRAF^V600E^-driven melanoma cells interconvert between two mutually exclusive states: a high^BRN2^ state linked to invasion and therapeutic resistance and a low^BRN2^ state associated with proliferation and tumor initiation.

### Bidirectional phenotype interconversion is orthogonal to cell cycle

Evidence for melanoma phenotype switching in prior studies has included the classification of single cells or populations of cells into subgroups based upon both gene expression and cell behavior, as well as the emergence of these subgroups in selective conditions, during tumor progression or upon molecular or genetic manipulation.^2,5,24,26,31,39^ However, the kinetics of phenotype switching in homeostatic conditions, including how switching is related to cell growth and division, remain largely uncharacterized.

**Figure 3.**
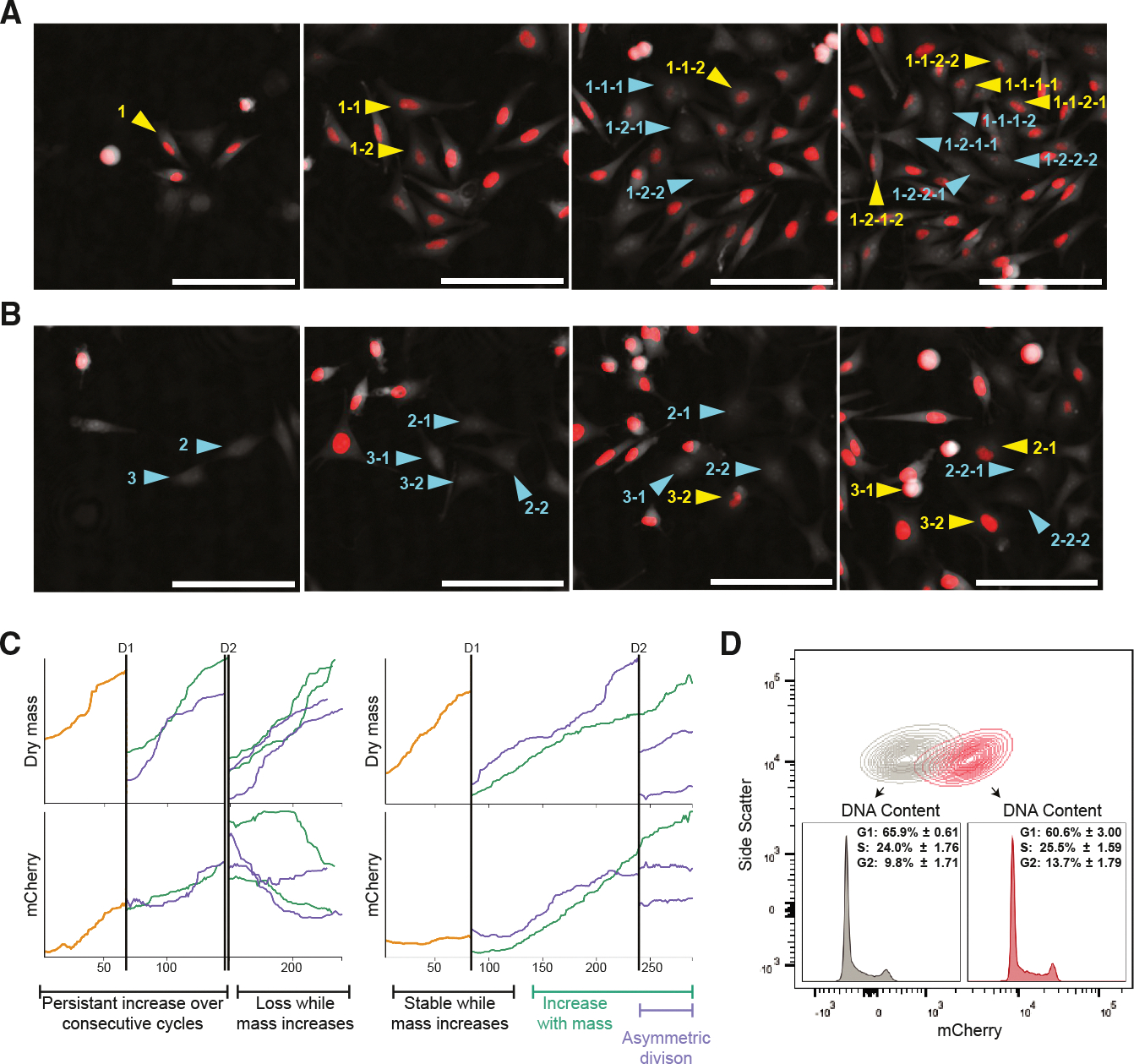
Melanoma cells undergo spontaneous bidirectional interconversion between BRN2-mCherry high and low states in a cell cycle-independent manner (A and B) Stills from representative Videos S1 and S2, respectively, taken every 30 min for 2 days. Parental cells (P1, P2, and P3) are highlighted in each (left column) and tracked through one or more divisions with yellow (high^BRN2^) and blue (low^BRN2^) arrow heads. Labels indicate lineage (e.g., P-F1-F2-F3). Scale bars, 200 μm. (C) Plots of relative total dry mass and total mCherry intensity normalized to the total dry mass of two example cell lineages through two divisions. The gold line indicates P0 parental cells, and the purple and green lines indicate the F1 daughter cells and the associated F2 progeny. In both lineages, total mass undergoes the expected cyclic doubling during cell growth and halving during cell division (top row). Changes in relative mCherry expression are neither cyclic nor always affected by division (bottom row). Examples of different patterns relating relative mCherry expression to total mass are annotated underneath. (D) DNA content of purified mCherry low and mCherry high cells. The percentage of cells in each cell cycle phase is shown as the mean and standard deviation of three biological replicates. (%G1 *p* = 0.0671, %S *p* = 0.3258, and %G2 *p* = 0.1009; unpaired *t* test). Representative intensity of mCherry and DNA profiles (insets) is shown.

We again employed ptychography coupled with fluorescence microscopy to monitor heterogeneous 624-mel cultures expressing the *BRN2*-mCherry reporter. We tracked the lineages of cells identified at time point zero and monitored mCherry expression level and cell morphology^53,54^ (Figures 3A–3C and S3). Overall, mCherry expression was stable in most cells over multiple divisions, such that the mCherry expression level of the parental cell was usually inherited by each daughter cell, resulting in clusters of either high^BRN2^ or low^BRN2^ cells (Videos S1 and S2). However, interconversions in both directions were also captured. For example, we observed high^BRN2^ parental cells giving rise to both high^BRN2^ and low^BRN2^ daughter cells (Figure 3A, cells 1–1, 1–1-1, and 1–1-2), and the subsequent reverse transition of low^BRN2^ progeny back to a high^BRN2^ phenotype (cells 1–1-1, 1–1-1–1, and 1–1-1–2). To assess whether *BRN2* expression oscillates with the cell cycle, we monitored the total integrated mCherry fluorescence from one M-phase to the next for each cell. M-phases are readily identified using quantitative phase imaging by the distinctive and characteristic morphologic pattern—a rapid increase in cellular sphericity coupled with halving of cell mass^54^ (Figures S3A and S3B). When adjusted for total cell mass, we observed no discernible pattern in mCherry expression that correlated with cell cycle (Figure S3C). When observing the lineages that begot changes in mCherry status, we observed multiple and inconsistent patterns of mCherry expression in relation to cell divisions (Figure 3C). These included a gradual increase in expression over consecutive cell divisions, loss of or stable expression even as total mass accumulated, and divisions that resulted in asymmetric mCherry expression. To further explore the relationship between *BRN2* expression and cell cycle, we purified high^BRN2^ or low^BRN2^ cells and conducted cell cycle profiling based on DNA content. Although cells with low *BRN2* expression exhibited a trend toward a higher percentage in G1 and a reduced percentage in G2/M, these differences were not statistically significant (%G1 *p* = 0.0671, %S *p* = 0.3258, and %G2 *p* = 0.1009, unpaired *t* test) (Figure 3D). These observations confirm that sporadic bidirectional phenotype switching occurs in human melanoma cells in homeostatic conditions. The kinetics of switching are orthogonal to the cell cycle, such that the two phenotypes can be inherited and typically remain stable through multiple cell divisions.

### BRN2 and MYC phenotypes interconvert through MITF states

**Figure 4.**
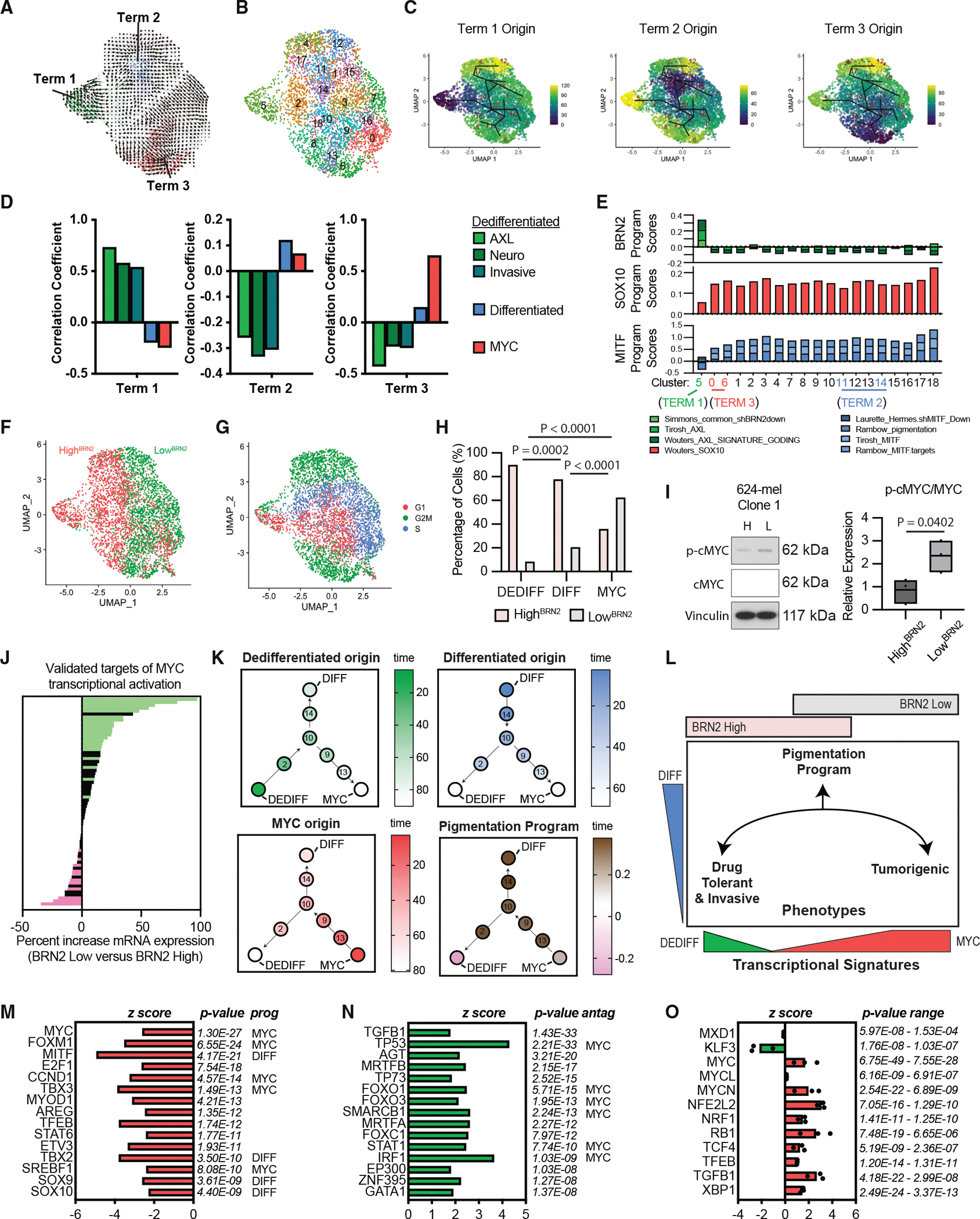
BRN2 and MYC phenotypes interconvert through MITF states (A–C) Uniform manifold approximation and projection (UMAP) visualization of 4,451 transcriptional profiles from a heterogeneous culture of low^BRN2^ and high^BRN2^ 624-mel human melanoma cells that passed quality control. Overlaid are: (A) RNA velocity vector field predictions, illustrating inferred directional transitions and convergence toward transcriptional terminal states (terms 1–3, colored); (B) Seurat-based clustering of cells into distinct transcriptomic populations; and (C) pseudotime trajectory analysis, with pseudotime values (represented as a color gradient) and predicted lineage trajectories originating from term 1–3. Individual pseudotime values for clusters along the predicted transitions between termini are plotted in Figure S4A. (D) Correlation coefficients of terms 1–3 with published transcriptional signature clusters (AXL, neuro, invasive, DIFF, and MYC). Positive scores indicate a higher correlation between the terminus and the specified transcriptional signature cluster, while negative scores denote anticorrelation. The transcriptional signatures used in this analysis and the dendrogram of clustered signatures are presented in Figure S4B. (E) BRN2/AXL, SOX10, and MITF program scores for Seurat clusters and terms 1–3. Signatures from Simmons 2017, Tirosh 2016, Wouters 2020, Rambow 2018, and Laurette 2014. (F–G) UMAP overlaid with: (F) mCherry FACS isolation tag (high^BRN2^ and low^BRN2^); and (G) cell cycle phase assignment. Additional overlays with signature enrichments are shown in Figures S4C and S4D. (H) The percentage of each terminus constituted by high^BRN2^ (pink) or low^BRN2^ (gray) cells. *p* values represent statistical significance from pairwise chi-square tests comparing the distribution of high^BRN2^ and low^BRN2^ cells across the termini. (I) Western blot analysis of cMYC and phospho-cMYC in FACS-enriched high^BRN2^ [H] and low^BRN2^ [L] cells. The graph indicates the expression of phospho-cMYC relative to the amount of cMYC. The mean protein expression, standard deviation, *p* value determined by standard *t* test, *n* = 3, independent experiments. Additional cell lines are shown in Figure S4E). (J) Comparative mRNA expression in high^BRN2^ versus low^BRN2^ cells for validated targets of MYC transcriptional activation. Red and green bars indicate significant enrichment (adjusted *p* < 0.05 by standard *t* test) in high^BRN2^ and low^BRN2^ cells, respectively. (K) Colorimetric depiction of cell state transitions that are dependent on assigned origins over time (summarized from pseudotime analysis shown in Figure S4A). Bottom right, juxtaposed colorimetric summary of the pigmentation program from Figure S4D. (L) Schematic summary of the relationship between observed transcriptional termini (DIFF, DEDIFF, and MYC), observed phenotypes (drug-tolerant and invasive, pigmentation program, and tumorigenic), as well as reporter status (BRN2 High and BRN2 Low). (M–O) IPA upstream regulator predictions (bias-corrected *Z* and overlap *p*). (M) Regulators predicted inhibited upon BRN2 overexpression. Regulators linked to MYC activity or melanocyte differentiation/pigmentation (DIFF) are annotated. (N) Regulators predicted activated upon BRN2 overexpression. Regulators with prior evidence of antagonism to MYC are annotated. (O) Regulators significantly altered across all three cell lines following pharmacologic BRN2 inhibition (B18–94). Each point represents a cell line comparison with the minimum and maximum overlapping *p* values observed across the three comparisons displayed. Only regulators significant in all lines are shown.

We next sought to explore the transcriptional profiles of cells with high and low *BRN2* expression and the transcriptional pathways of their state interconversions. We conducted single-cell RNA sequencing (scRNA-seq) on a heterogeneous culture of low^BRN2^ and high^BRN2^ 624-mel cells containing, as established above, a mixture of both stable and transitioning cells. Low^BRN2^ and high^BRN2^ cells were first isolated via FACS and molecularly tagged prior to analysis. RNA velocity analysis leverages spliced and unspliced mRNA abundances to infer transcriptional trajectories, predicting terminal states where cells stabilize and intermediate cells transitioning between these termini.^55,56^ This approach predicted three transcriptional termini (Figure 4A, terms 1–3). To predict the likely pathways by which cells traverse to each terminus, we conducted pseudotime analysis—a method for ordering the transcriptional profiles of single cells to provide a probabilistic reconstruction of cell-state transitions.^57^ Seurat clustering at resolution 2 identified nineteen clusters (Figure 4B), and pseudotime analysis was initiated from each of the three termini. In all cases, the inferred trajectories connecting the three termini passed through the same shared intermediate clusters (Figures 4C and S4A).

To benchmark the transcription programs associated with each termini against well-documented cell state profiles, we first implemented a classification correlation analysis that clusters gene signatures.^58^ Term 1 clustered with signatures associated with dedifferentiation—including the original Hoek 2006 invasive signature,^59^ neural crest signatures, and healthy primary human melanocyte stem cell signatures—and most closely with the Tirosh 2016 AXL signature^35^ (Figures 4D, S4B, and S4C). Term 1 also had the highest expression of BRN2-regulated genes^31^ and AXL-regulated genes^3,35^ (Figure 4E). We therefore refer to this terminus as dedifferentiated (“DEDIFF”). Term 3 clustered with signatures associated with DNA replication and mitosis that are significantly enriched for MYC targets^58^ (Figures 4D and S4B). We refer to this terminus as MYC/mitosis (“MYC”). All other clusters, regardless of the reporter status and inclusive of term 2 and transition states, expressed SOX10^3^- and MITF-regulated genes,^4,35,60^ including pigmentation genes^4^ (Figures 4E, S4C, and S4D). Term 2 itself clustered with signatures of differentiation, including the original Hoek MITF signatures, pigmentation signatures, and healthy human adult melanocyte signatures (Figures 4D, 4C, and S4B–S4D), and is therefore referred to as differentiated (“DIFF”).

To determine how the BRN2 reporter status corresponded to each terminus, we next considered the mCherry status of each cell. Uniform manifold approximation and projection (UMAP) revealed distinct clustering of low^BRN2^ and high^BRN2^ cells, affirming their close relationship yet clear separation (Figure 4F). Cell cycle phase assignment did not correspond to state-specific clusters, re-emphasizing the distinct and orthogonal nature of cell cycle dynamics from BRN2 status (Figure 4G). Notably, the DEDIFF terminus was composed predominantly of high^BRN2^ cells, the MYC terminus predominantly of low^BRN2^ cells, and the DIFF term an intermediate percentage of each (Figures 4H and S4F). Consistent with these observations, sorted high^BRN2^ cells expressed more AXL protein than low^BRN2^ cells (Figure S4G), and sorted low^BRN2^ cells expressed more phosphorylated MYC protein (Figures 4I and S4E) and significantly elevated expression of transcripts of validated targets of MYC^61^ (Figure 4J). Though the DEDIFF and MYC termini were the only clusters with reduced expression of pigmentation-associated genes (Figures 4K and S4D), since a comparable and sizable fraction of both low^BRN2^ and high^BRN2^ cells occupied MITF-expressing states (Figure S4F), we were unsurprised to observe little difference in MITF expression between the respective purified cells (Figure S4G). Thus, while BRN2 expression reliably distinguishes cells in DEDIFF versus MYC-driven states, it fails to segregate the MITF-high intermediate and terminal states, which represent the majority of cells in both populations (summarized in Figure 4L).

Our findings suggest that human melanoma cell lines can adopt two transcriptomically and phenotypically distinct, mutually exclusive “MITF-low” states—DEDIFF and MYC. To explore this model across multiple lines and minimize the possibility that the observation was an artifact of gene dropout, we performed high-coverage scRNA-seq of seven clonal populations: 624-mel (*n* = 134, 667, 494, and 493), SK-MEL-28 (*n* = 660, 537), and WM793 (*n* = 220) with a median detected genes per cell of 9,643. Although the number of cells per clone was below the threshold typically required for standard clustering, this high-coverage design provided confidence in gene expression analyses. We computed Pearson correlations between the DEDIFF and MYC programs for each melanoma cell line. All lines exhibited a significant negative correlation (r = −0.196, −0.244, and −0.101; *p* < 0.001 for all comparisons, Table S1), indicating that the co-activation of DEDIFF and MYC programs is rare. Consistent with our model, this anticorrelation was most pronounced in cells with uniformly low differentiation program activity. In low-DIFF cells, the correlations between DEDIFF and MYC were r ≈ −0.888, −0.905, and −0.992, much stronger than in high-DIFF cells, indicating a sharper trade-off between these two alternative programs under minimal differentiation (Table S2). In short, the DEDIFF-MYC axis was pronounced in cells lacking the differentiation program, whereas in cells where the differentiation program is active, the direct DEDIFF-MYC anticorrelation becomes less apparent.

To assess whether BRN2 expression suppresses MYC activity, we reanalyzed two recently published datasets. In the first, RNA expression profiling was performed in three BRAF^V600E^ melanoma cell lines (MM370, MM603, and MM455) harboring doxycycline-inducible BRN2.^62^ Transcriptomes with and without BRN2 induction were compared, and Ingenuity Pathway Analysis (IPA, Qiagen) “upstream regulator” inference was applied. The most significantly inhibited transcriptional regulators associated with BRN2 overexpression were MYC and its target FOXM1 (Figure 4M), with additional predicted inhibition of MYC targets and MYC-associated regulators (TBX3, CCND1, and SREBF1). The regulators of melanocyte differentiation/pigmentation (MITF, TBX2, SOX9, and SOX10) were likewise predicted to be inhibited, consistent with BRN2 opposing both the DIFF and MYC programs. Similarly, 6 of the top 15 predicted activated regulators under BRN2 overexpression are established antagonists of MYC signaling (Figure 4N).

In the second study, three additional BRAF^V600E^ melanoma lines (C32, MM383, and MM386) were profiled by quantitative mass spectrometry following treatment with B18–94, a small-molecule BRN2 inhibitor that disrupts BRN2-DNA binding.^63,64^ IPA applied to protein-level changes yielded concordantly inverse predictions: among regulators consistently affected across all three lines, MXD1 (a canonical MYC antagonist) was predicted to be inhibited upon BRN2 inhibition, whereas MYC was the top predicted activated regulator, with MYCL and MYCN also activated (Figure 4O). Taken together, BRN2 overexpression is associated with reduced predicted MYC activity, whereas pharmacologic BRN2 inhibition is associated with increased predicted MYC activity. Collectively, these convergent analyses support a BRN2-MYC antagonistic relationship across the nine BRAF^V600E^ melanoma cell lines examined.

### Transformed melanocytes exhibit reduced phenotypic stability and enhanced access to the MYC state

Melanoma cells are widely recognized for their high phenotypic potential and plasticity.^2–5^ However, it remains less explored whether these attributes are exclusive to cancer cells or also inherent to healthy melanocytes. Primary melanocytes from discarded neonatal foreskin are typically used as the non-transformed reference for melanoma cells. However, the transcriptional programs of neonatal foreskin melanocytes fundamentally differ from those originating from adult skin.^36^ Primary human melanocytes also exhibit significant plasticity in culture when exposed to various growth factors,^42,43^ but the rate of spontaneous phenotypic switching under homeostatic conditions, as observed in this study for melanoma cells, remains undefined. We thus aimed to determine if the distinct cell states and interconversions noted in melanoma cells are also present in non-transformed melanocytes present in human skin.

**Figure 5.**
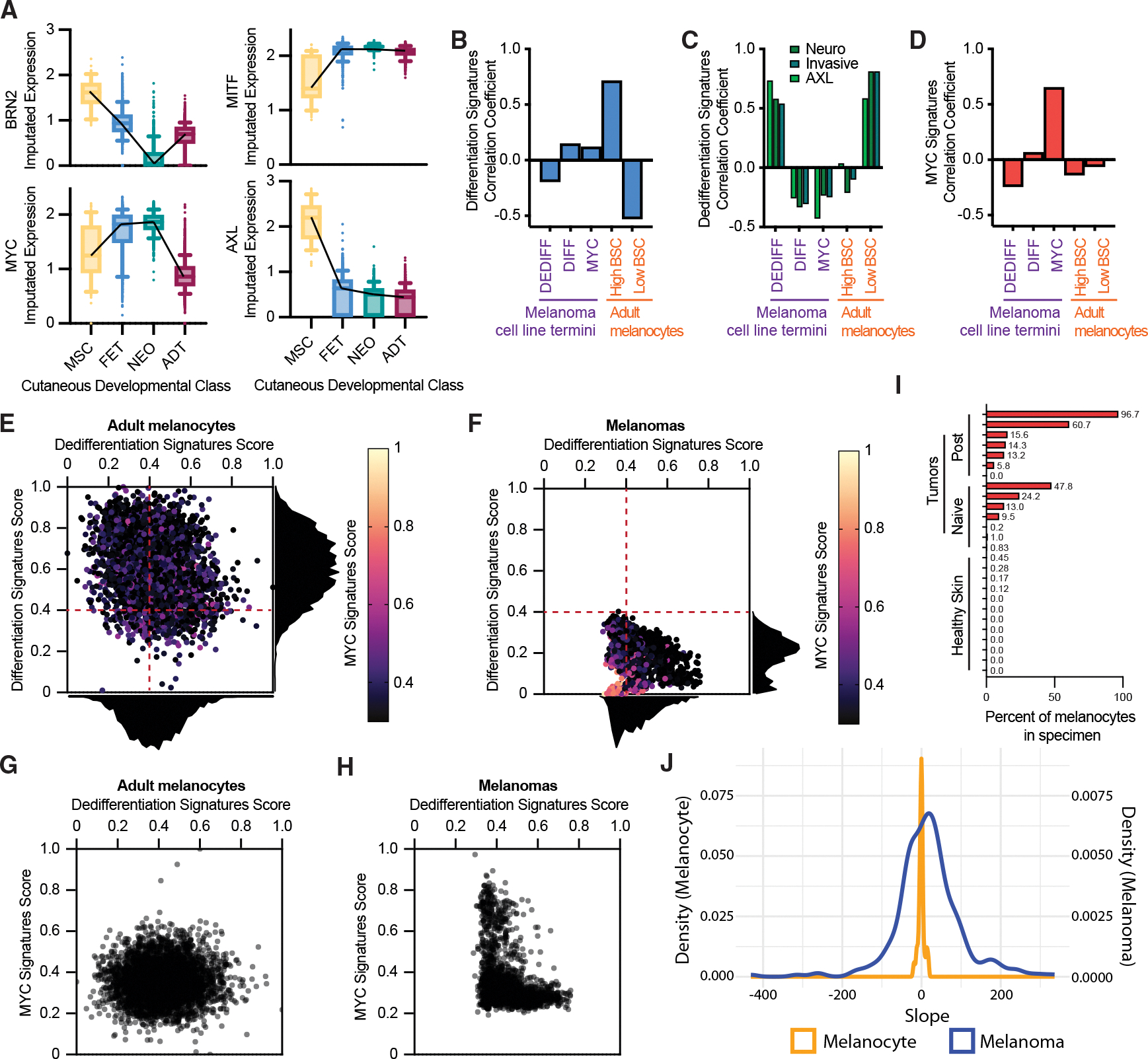
Spontaneous interconversions and the MYC state are unique to transformed melanocytes (A) Comparative expression of BRN2 and MYC between melanocyte stem cells (MSC), fetal melanocytes (FET), neonatal foreskin-derived melanocytes (NEO), and adult cutaneous epidermal melanocytes (ADT) from healthy skin. Tukey style box and whisker plot, boxes indicate interquartile range (IQR) with center line at median, whiskers extending 1.5 IQR, *n* = 63 (MSC), 1,176 (FET), 735 (NEO), and 3,280 (ADT). (B–D) Correlation coefficients of melanoma cell line termini (from Figure 4D) and adult melanocyte high and low BSC populations (Figure S5B) with published transcriptional signature clusters (AXL, Neuro, invasive, DIFF, and MYC) using WIMMS (Hu, 2024^58^). Positive scores indicate a higher correlation between the terminus and the specified transcriptional signature cluster, while negative scores denote anticorrelation. (E–H) Comparative analysis of the signature scores from scRNA-seq datasets examining relationships between expression programs in healthy skin melanocytes and melanoma cells. (E and F) Dedifferentiation versus differentiation scores, with MYC scores represented as a color gradient for adult melanocytes (E) and melanoma cells (F), and histograms shown along the axes. Dotted red lines are included as reference markers to facilitate comparison of the distributions. (G and H) Dedifferentiation versus MYC scores for adult melanocytes (G) and melanoma cells (H). (I) Classification of melanocytes based on correlation with the MYC program, categorized into healthy skin (percent MYC-state melanocytes per adult skin specimen), treatment-naïve tumors, and post-treatment resistant tumors (percent MYC-state melanocytes per tumor specimen). (J) Density plot showing the distribution of normalized mCherry expression rate changes over time for melanocytes (LP051, orange, *n* = 56) and melanoma cells (624-mel clone 1, blue, *n* = 301). Positive slopes represent transitions toward the high^BRN2^ state, while negative slopes indicate transitions toward the low^BRN2^ state. Examples of individual cells are shown in Figures S6A and S6B.

Initially, we evaluated melanocytes at different stages of development for the expression of key genes identified in this study (*BRN2*, *MYC*, *MITF*, and *AXL*), utilizing our established scRNA-seq database of epidermal melanocytes directly sorted from fresh, healthy human skin.^36^ Given that transcription factors and surface proteins are underrepresented in scRNA-seq datasets, we employed an imputation pipeline to assess their expression patterns.^65^ Our ensuing observations aligned with previous findings while also providing insights into the nuances of *BRN2* expression during human melanocyte development. For example, high *BRN2* expression was observed in melanocyte stem cells and fetal melanocytes and was absent in neonatal foreskin-derived melanocytes (Figure 5A, upper left graph, compare columns 1 and 2 with 3), consistent with prior literature.^66^ However, to our surprise, adult cutaneous epidermal melanocytes re-expressed the transcription factor (Figure 5A, upper left graph, compare columns 3 and 4). Similarly, we recapitulated the previously reported inverse relationship between *BRN2* and *MITF* expression, but only when comparing melanocyte stem cells to non-stem melanocytes (Figure 5A, upper graphs, compare columns 1 and 2); this inverse expression pattern did not hold when comparing distinct populations of DIFF melanocytes (compare columns 2–4). In contrast, two sets of genes—*MITF*-*AXL* and *BRN2*-*MYC*—displayed inverse patterns across all developmental stages (Figure 5A, upper left versus lower left and upper right versus lower right). These observations further support the interpretation that while BRN2 and MITF may function as a bistable transcriptional switch in stem-like versus DIFF populations, two distinct dichotomous switches—MITF-AXL and BRN2-MYC—are more consistent within the melanocytic lineage across all stages of development.

Intrigued by the unexpected expression of *BRN2* in adult melanocytes, we next investigated whether adult epidermal melanocytes exhibit transcriptional diversity comparable to that observed in melanoma cells. UMAP analysis Seurat clustering of adult melanocytes identified seven distinct clusters (Figure S5A). To assess pigmentation, we overlaid backscatter (BSC) data, a feature correlating with melanin content,^36,67^ and identified two distinct populations: one with higher BSC values and one with lower BSC values (Figure S5B). Pseudotime analysis supported the potential transitioning between these two distinct populations (Figure S5C). All adult skin specimens contained cells from both populations (Figure S5D). Classification correlation analyses of genes differentially expressed between the two populations confirmed that pigmented melanocytes (high BSC) closely correlated with DIFF signatures (Figure 5B). Notably, while the DIFF terminus of melanoma cells also correlated with differentiation signatures (Figure 4D), the correlation was less pronounced than for high BSC melanocytes (Figure 5B), supporting the interpretation that “MITF high” or “DIFF” melanoma cells do not express these programs as robustly as non-transformed adult pigmented melanocytes. In contrast, low BSC melanocytes correlated as closely with dedifferentiation programs as their melanoma counterparts, exhibiting a pattern strikingly similar to the DEDIFF subset of high^BRN2^ cells (Figure 5C). Neither healthy skin melanocyte population exhibited a correlation with MYC signatures (Figure 5D).

To further explore the relative levels and frequency of differentiation, dedifferentiation, and MYC signatures in healthy melanocytes versus melanoma cells, we calculated a classification score for each cell in the adult melanocyte scRNA-seq dataset from Belote et al.,^36^ and each malignant cell from fourteen *ex vivo* human melanoma tumors generated by Tirosh et al.^35^ and Jerby-Arnon et al.^37^ Although healthy adult melanocytes generally expressed high levels of differentiation signatures, there was a broad distribution that inversely correlated with the expression of dedifferentiation signatures (Figure 5E). This inverse correlation was also evident in melanoma cells (Figure 5F). Consistent with the above observations, the expression of differentiation signatures in malignant cells was uniformly less robust compared to healthy melanocytes, whereas the distribution of dedifferentiation signatures was comparable between healthy and transformed cells (compare dotted red lines and histograms in Figures 5E and 5F). Another notable distinction between the two populations was the presence of a MYC program-expressing population that expressed neither the dedifferentiation nor differentiation signatures and was exclusive to the malignant population (Figures 5E and 5F, colorimetric; and G and H, *y* axis). We classified each cell based on its predominant correlation, finding that few melanocytes in healthy skin exhibited MYC program dominance, whereas most melanomas (10 of 14) contained a population of MYC cells at 5% or greater (Figure 5I).

We were surprised to observe that healthy skin contains epidermal melanocytes with dedifferentiation signatures as robust as those found in melanomas. We examined whether primary human melanocytes, too, oscillate between these cell states like melanoma cells. We transduced the BRN2-mCherry reporter into low-passage primary human melanocyte cultures and similarly performed ptychography coupled with fluorescent microscopy to monitor fluctuations in BRN2 expression. The intensity of mCherry relative to cell mass was monitored over time for each tracked cell (examples in Figures S6A and S6B). Healthy melanocytes displayed stable mCherry expression, with minimal variation in either direction, whereas melanoma cells exhibited fluctuations in mCherry expression, including both gains and losses (Figures 5J and S6A–S6C). The stability of mCherry expression across different primary melanocyte preps (*n* = 4) as compared to different melanoma lines (624-mel, 4 clones and SK-MEL-28, 2 clones) remained consistent (*p* = 2.2E-16) even during extended imaging durations (Figure S6D). These findings support a model where healthy melanocytes express both DIFF and DEDIFF programs commonly associated with advanced disease, yet do not show the phenotypic volatility observed in melanoma cells.

## DISCUSSION

In this study, we used label-free live cell imaging coupled with a fluorescent reporter of *BRN2* promoter activity to monitor the kinetics of phenotype switching in primary human melanocytes and melanoma cells in homeostatic conditions. Our work builds upon prior studies that applied chemical, environmental, or genetic perturbation, then indirectly inferred phenotype switching by taking molecular snapshots at different time points. We demonstrate phenotype heterogeneity within clonal populations that is re-established in standard culture conditions, as has been previously demonstrated for breast cancer and glioblastoma.^68,69^ Switching occurs on an “intermediate time-scale,”^70^ such that phenotypes are inherited through multiple generations while also undergoing spontaneous interconversion. Since switching was bidirectional, our observations reinforce the dynamic stemness model in melanoma,^6^ wherein tumorigenic cells arise spontaneously from non-tumorigenic cells and vice versa to reach a phenotypic equilibrium. It is notable that each of the mutually exclusive transcriptomic states studied here exhibited separate tumor-associated phenotypes. Our data suggest that once a melanoma is established, the induction of either state could progress the disease in separate ways, increasing tumorigenicity or decreasing therapeutic sensitivity. The precise equilibrium is likely dependent on both cell intrinsic and extrinsic factors, which were not explored in this study.

Pioneering work in this domain identified two melanoma phenotypes—an MITF-high and BRN2-low proliferative state and an MITF-low and BRN2-high invasive state.^23–25,59^ Our observations are consistent with a more complex phenotypic landscape.^3,4,22,28,31,34,71^ First, we observed that the reported MITF-BRN2 bistable switch is present when comparing melanocyte stem cells to DIFF melanocytes. However, when comparing distinct DIFF populations of melanocytic cells, including melanoma cells, the MITF and BRN2 programs appear orthogonal, such that “BRN2 high” populations can contain both “MITF high” and “MITF low” cells. Instead, our data support the existence of two switches in melanocytic cells: MITF-AXL and BRN2-MYC. It is the latter that the presented reporter identifies. The poles of this BRN2-MYC spectrum are likely similar to the BRN2 and SOX10 states identified in earlier studies.^4–6,22,40^ Consistently, BRN2 melanoma cells have been associated with increased tolerance to targeted therapy, whereas SOX10 cells were depleted in drug-resistant tumors.^4,5^ The mutual exclusivity of these two states, yet their individual contributions to different stages of melanoma progression (i.e., tumor initiation versus therapeutic resistance), offers a rational explanation for the paradoxical identification of BRN2 as both an oncogene and a tumor suppressor in prior studies.^26,38,39^

Among our more surprising observations is the identification of epidermal melanocytes in healthy adult human skin associated with signatures of dedifferentiation. It is important to emphasize that these cells were not associated with the MYC program that uniquely marked the tumorigenic melanoma cells. Thus, one feature unique to melanoma cells is the expanded phenotype repertoire to include this tumor-initiating state. A second, distinct feature is the expanded ability of melanoma cells to switch between phenotypes. This difference in transcriptomic instability, independent of phenotype potential, likely renders melanoma cells more adaptable to fluctuating environments.

Overall, this research provides useful insights into the dynamics of phenotype switching in melanoma cells. Our findings highlight the importance of understanding the mechanisms underlying phenotypic switching in melanoma and the relationship between therapy-tolerant and tumorigenic properties. We emphasize the implications of our observations for strategies seeking to enhance the efficacy of melanoma therapies by targeting or inducing specific phenotypes. As the induction of either phenotype characterized here could be detrimental to a patient, future studies should weigh the potential benefit of sensitizing cells to therapeutic regimens against the potential harm of increasing the risk of metastatic outgrowth and vice versa.

## RESOURCE AVAILABILITY

### Lead contact

Further information and requests for resources and reagents should be directed to and will be fulfilled by the lead contact, Robert L. Judson-Torres (robert.judson-torres@hci.utah.edu).

### Materials availability

This study did not generate new, unique reagents.

### Data and code availability

- mRNA-sequencing data produced in this manuscript are available from Gene Expression Omnibus (GEO) and accessible by entering the following GSE accession numbers into the search bar: GEO: GSE150582 and GSE230574.
- This paper does not report original code.
- This paper does not report any additional resources.

## STAR★METHODS

### EXPERIMENTAL MODEL AND STUDY PARTICIPANT DETAILS

#### Human tissue procurement and ethical approval

Healthy adult human skin specimens were obtained under a University of Utah Institutional Review Board–approved tissue collection protocol (IRB #89989) administered through the Huntsman Cancer Institute (HCI). Donors provided written informed consent prior to tissue collection, and all specimens were fully de-identified before delivery to the laboratory. Four independent donor skin samples (*n* = 4) were used in this study. Tissue collection occurred without regard to donor sex, ethnicity, or race, and no personally identifiable information was accessible to investigators. Human skin was used exclusively to derive primary melanocyte cultures for downstream experimental assays; therefore, donor-level assignment to control or experimental groups was not applicable.

#### Cell culture maintenance and manipulation

Melanoma cell lines 624-mel (CVCL_8054), SK-MEL-28 (CVCL_8054) and WM793 (CVCL_8787) were received as gifts from Dr. Boris Bastian and Dr. Meenhard Herlyn. Lines were STR verified (Table S3) and regularly monitored with the Universal Mycoplasma Detection Kit (ATCC 30–1012K). Cultures were maintained in base media (RPMI1640, Thermo Fisher, 11–875-093, DMEM, Thermo Fisher, 11–965-092, or Ham’s/F12, Thermo Fisher, 11–550-043) supplemented with 10% FBS (Corning, 35–010-CV), 1× penicillin-streptomycin (Thermo Fisher, 15140122), and 2mM L-glutamine (UCSF core facility, CCFGB002) at 37°C, 5% CO_2_. All cells were disassociated with 0.05% Trypsin EDTA (Mediatech, MT 25–052-C1) followed by neutralization with equimolar soybean trypsin inhibitor (Thermo Scientific, 17075029). Primary human melanocytes were isolated from de-identified and IRB consented neonatal foreskins or adult skin. Skin tissue was incubated overnight at 4°C in dispase followed by removal of epithelia. Epithelial tissue was then minced and incubated in 0.25% trypsin (Gibco, 25200056) for 4 min at 37°C. Trypsin was quenched and tissue was centrifuged at 500 × g for 5 min at room temperature. The pellet was resuspended in melanocyte medium (Thermo Fisher Scientific, M254500) containing HMGS (Thermo Fisher Scientific, S0025) and plated. The BRN2 reporter (pROM-POU3F2p-mCherry-Neo, Addgene 153321) was engineered by first inserting NLS-mCherry (Gift from Ron Vale, Addgene 67932) and a pGK-driven neomycin resistance cassette into a 3^rd^ generation vector backbone, pSicoR-Ef1a-mCh-Puro, (Gift from Bruce Conklin, Addgene 31845).^73,74^ The BRN2 promoter was amplified from human DNA (key resources table). Lenti-viral particles were generated and transduced at MOI <0.3 in the presence of 10 μg/mL polybrene (Sigma TR-1003).^38^ Three days later, pROM-POU3F2p-mCherry-Neo transduced cells were selected with neomycin (Sigma, A1720) for 3 weeks at the minimal concentration required for complete toxicity of parental cells (cell line dependent, concentrations ranged from 100 μg/mL to 1000 μg/mL). Single cells from each selected culture were sorted using the SONY SH800 FACS machine and expanded. Neomycin was periodically added to the media to ensure retention. For isolation experiments, respective populations were FACS isolated twice in tandem followed by a third analyses to ensure >99% purity. Flow analyses were conducted with either a Sony SH800 or BD Fortessa.

#### Maintenance and care of mice

All animal work was conducted under protocols approved by the University of Utah Institutional Animal Care and Use Committee (IACUC; Protocol #00002405 and in accordance with the Guide for the Care and Use of Laboratory Animals (8th edition, 2011) and the Animal Welfare Act. The University of Utah is fully accredited by the American Association for Accreditation of Laboratory Animal Care (AAALAC) and maintains a Public Health Service (PHS) Animal Welfare Assurance. Veterinary oversight and husbandry were provided by the Office of Comparative Medicine (OCM).

Eight-week-old male and female NOD.Cg-Prkdcscid Il2rgtm1Wjl/SzJ (NSG; The Jackson Laboratory, stock no. 005557) mice were used for all studies. Animals were housed in the Huntsman Cancer Institute (HCI) specific-pathogen-free (SPF) vivarium managed by the Preclinical Cancer Models Shared Resource (PCM). Mice were maintained in individually ventilated cages (IVCs) with HEPA-filtered air supply and independent exhaust. Environmental conditions were controlled at 22 ± 2°C, 40–60% relative humidity, and a 12-h light/dark cycle. Autoclaved bedding, irradiated rodent chow, and water were provided *ad libitum*.

Environmental enrichment, including nesting material and shelters, was provided to support normal behavior and reduce stress. Mice were group-housed whenever compatible with study design and behavior. Body weight and condition were recorded routinely, and animals exhibiting clinical signs of illness or distress were promptly evaluated by veterinary staff. Humane endpoints were applied as defined by institutional policy to minimize pain and discomfort.

### METHOD DETAILS

#### ATAC-seq

The ATAC-seq assay followed the published Omni-ATAC protocol.^75^ Nuclei were isolated from 50000 cells, which were then treated with 2.5 μl of Tn5 transposase (Illumina 20034197) for DNA tagmentation. DNA was extracted and PCR amplified (5 cycles) using barcoded primers that were included in the Omni-ATAC protocol.^75^ The ATAC libraries were sequenced by NovaSeq (50bp paired end). ATAC-sequenced reads were quality checked by FastQC (v0.11.9), and then aligned to the human reference genome hg19 (GRCh37) using Bowtie2 aligner (bowtie2-align-s version 2.4.2). Utilizing samtools (version 1.12), all mitochondrial, unaligned, and low-quality reads (Q < 20) and duplicated reads were filtered out, and were then normalized for equal coverage in each group of cells. Removing adapters by NGmerge (version 0.3), ATAC-enriched regions/peaks were identified by MACS (3.0.0a6) with Q value cut off of 0.01 and with other default parameters. The identified peaks were visualized through Integrated Genome Browser (IGB), and also were annotated by HOMER (v4.11.1-the annotatePeaks.pl program). Finally, the differentially enriched peaks between two groups of cells were evaluated via DiffBind (version 3.4.3), using DESeq2 method.

#### Protein preparation and western blotting

At least 200,000 cells were lysed in RIPA buffer (Thermo 89901) containing a protease inhibitor cocktail (Thermo 87785) and 0.5 M EDTA (Thermo 1861274), each used at 1:100 concentration, incubated on ice for 10 min, then centrifuged at maximum speed for 20 min at 4°C. The protein concentration of the supernatant was measure using the Pierce BCA protein assay kit (Thermo 23225) and 10 μg of protein was loaded on a 4–12% Nupage bis-tris gel (Thermo NP0323box), run for 10 min at 50 V and 1.5 h at 100 V in NuPAGE MOPS SDS Running buffer (Thermo NP0001), and dry-transferred onto a PVDF membrane (Thermo IB24001) using an Invitrogen iBlot 2 Gel Transfer Device (Invitrogen IB21001). The membrane was submerged in 5% milk dissolved in TNET (1M Tris pH = 7.4, 0.5M EDTA, NaCl, Tween, diH_2_O) for 1 h before incubation with a primary antibody diluted in 5% BSA dissolved in TNET (key resources table) overnight, followed by trice washing in TNET before a secondary antibody (Invitrogen G-21040 or Invitrogen G-21234, 1:10000) was added and incubated for 1 h. After three washes, the membrane was imaged using an Azure Biosystem Imager. The band intensity was measured by densitometry using ImageJ 1.52a.

#### Quantitative real-time PCR

FACS isolated cells were added to 0.5–1 mL of Trizol (Ambion 15596026) for RNA extraction. Growth media was removed and cells washed with PBS. 1 mL of Trizol reagent was added per 1 × 10^6^ cells directly into the cell dish – then subsequently pipetted up and down to homogenize. Samples were incubated for 5 min and then 0.2 mL of chloroform (Sigma Aldrich 472476) was added per 1 mL of Trizol reagent, then thoroughly mixed and followed by another 3-min incubation period. Samples were centrifuged at 12,000 × g at 4°C for 15 min to separate phenol-chloroform, interphase, and upper aqueous phases. Aqueous phase was isolated which contained the desired RNA for subsequent ethanol isolation. To precipitate RNA, 0.5 mL of isopropanol (Acros 67–63-0) was added per 1 mL of Trizol reagent followed by a 10-min incubation period. Samples were centrifuged for 10 min at 12,000 × g at 4°C to generate RNA precipitate. RNA was washed with 1 mL of 75% ethanol per 1 mL of Trizol reagent, vortexed, then centrifuged for 5 min at 7,500 × g at 4°C. Supernatant was discarded and the pellet was dried for 5–10 min at room temperature. The RNA pellet was then resuspended in RNase-free water containing 0.1 mM EDTA. For qRT-PCR, cDNA from 0.5 to 1 μg of RNA (NanoDrop, Thermo Scientific) was synthesized using the Sensifast synthesis kit (Bioline Bio-65053, manufacturer’s protocol) then diluted 1:5. Per RT-qPCR reaction, 1 μL of the dilution was mixed with 0.41 μM primers (key resources table) and 1× Sensifast SYBR non-Rox kit (Bioline Bio98005). The real-time PCR program cycled as follows: one cycle of 95°C for 2 min followed by 40 cycles of 95°C for 5 s and 65°C for 30 s. All primers and reagents can be found in the key resources table.

#### Single molecule RNA FISH

smFISH experiments were conducted with in-house reagents. Briefly, probes were developed using the designer tool from Stellaris (LGC Biosearch Technologies) using a masking level of 5, a minimum of 2 base pair spacing between single probes, and a length of 18 nt (key resources table). Approximately 5 × 10^5^ disassociated cells were immobilized on a Cell-Tak (Corning, CB-40240) coated 8-well chambered image dish, fixed with 5% formaldehyde (Tousimis 1008A), 1× PBS for 10 min, washed with 1× PBS then stored in 70% EtOH at 4°C for a minimum of one hour. Cells were then washed first with 2× SSC (Thermo Fisher Scientific 15557044) containing 10% v/v deionized Formamide (Thermo Fisher Scientific AM9342), then with hybridization buffer consisting of 10% w/w dextran sulfate (Sigma Aldrich 42867) in 1× SSC with 10% v/v Formamide, and incubated overnight at 37°C in hybridization buffer containing 25 nM probes. Cells were then incubated for 30 min at 37°C in 2× SSC containing 10% v/v Formamide, followed by 15 min at 37°C in 2× SSC containing 10% v/v Formamide and DAPI (Thermo Fisher Scientific D1306). Finally, cells were washes with 2× SSC and incubated for 2 min at room temperature in 2× SSC, 10% w/w glucose (Sigma Aldrich G7021), 0.01 M Tris pH 8 (Life Technologies AM9855G). To minimize photo bleaching, cells were imaged in a photo-protective buffer containing 2× SSC, 10% w/w glucose, 0.01 M Tris pH 8, 75 μg/mL glucose oxidase (Sigma Aldrich G0543–10K), 520 μg/mL catalase (Sigma Aldrich C3156–50), and 0.5 mg/mL Trolox (Sigma Aldrich 238813). Images were taken with a Nikon Ti-E microscope equipped with a W1 Spinning Disk unit, an Andor iXon Ultra DU888 1k x 1k EMCCD camera and a Plan Apo VC 100×/1.4 oil objective in the UCSF Nikon Imaging Center. Approximately 10 xy locations were randomly selected for each condition, and analyzed using Fiji and in-house programs.^72,76^

#### Quantitative immunofluorescence

Cells were fixed in 4% paraformaldehyde (PFA) in PBS (Biotium 22023) for 15 min at room temperature followed by pre-cooled (−20°C) methanol for 10 min at −20°C. Cells where twice washed in PBS then incubated in blocking buffer (5% Donkey Serum (Jackson Immuno Research Labs 017–000-121, 1% BSA (Sigma A9647), 0.1% Triton X-100 (Sigma T8787) in PBS) for one hour at room temperature. Cells were incubated at 4°C overnight with anti-BRN2 (Cell Signaling 12137S, 1:1000), thrice washed in PBS and incubated at room temperature for 1 h with goat anti-rabbit Alexa Fluor Plus 488 (Invitrogen, A32731, 1:10000). Cells were washed, incubated with DAPI (Thermo Fisher D1306, 1:1000) for 5 min at room temperature, washed twice more and imaged using the INCell Analyzer 2000 high throughput high content imager (GE).

#### Quantitative phase imaging to assess proliferation, motility, morphology and reporter fluorescence

100,000 cells were seeded per well of a standard tissue-culture treated 6-well polystyrene plate (Sarstedt 83.3920.005) for digital holographic cytometry (DHC) using the M4 Holomonitor (Phase Holographic Imaging, Sweden). Cells were monitored for 72 h. Morphology was analyzed using the HStudio Software and linear discriminant analysis.^44–46^ For Fourier ptychography coupled to fluorescent microscopy, 1,200 cells were seeded per well of a standard-culture treated 96-well plate then imaged with the LiveCyte (Phasefocus, United Kingdom) every 15–30 min for 48–72 h. Proliferation and motility were analyzed using the Analysis dashboards provided by the manufacturer. Cell mass was calculated by segmenting cell objects from background using Sobel edge detection thresholding, then summing the phase shift relative to background over all object (cell) pixels, and assuming a specific refractive increment of 1.8 × 10^−4^ m^3^/kg^51^. Cell lineages and masses were visualized using customized software Loon^53^ and Aardvark.^54^

Before calculating the normalized rate of change of the BRN2-reporter fluorescence intensity, the dataset was filtered by cell duration and dry mass. The duration of each cell was tracked by first converting frames to hours, and only cells tracked for at least 18 h were included in the analysis. Tukey’s fence outlier test was performed on the recorded dry mass values to filter out non-cell objects. The test was conducted separately for the two cell populations, and the standard outlier threshold (k = 1.5) was used. The number of dry mass outliers was calculated for each cell in the two populations, and tracks with at least one outlier were excluded from downstream analysis. After filtering, the recorded dry mass values were normally distributed. For each individual cell, the BRN2-reporter fluorescence integrated intensity was then normalized to its dry mass at every time point.

The normalized intensity versus time for each cell was plotted and fit to a linear regression model. For melanocytes, the coefficient of variation (standard deviation/mean) was −13.07691, and the index of dispersion (var/mean) was −94.27149. For melanoma, the coefficient of variation was 4.820381, and the dispersion index was 406.3269. Additionally, a two-sample Kolmogorov-Smirnov (KS) test was performed, yielding D = 0.48173 with a *p*-value = 6.081e-10, confirming that the normalized BRN2 intensity slopes of the two populations are not from the same distribution. Additionally, we compared the linear regression model (LM) to a linear mixed effects (LME) model. The LME model was fit to the normalized BRN2 intensity over time, where time was treated as a random effect to account for variability between cells. For each cell, LM and LME slopes were plotted against each other. Most cells fell on the 1:1 line, indicating that the random effect was negligible (Figure S5E).

#### Engraftment efficiency assay

All protocols described in this and other sections regarding animal studies were approved the Institutional Animal Care and Use Committees at UCSF, the University of Utah, and the Huntsman Cancer Institute. Ethical endpoint for tumor transplantation experiments was reached when a tumor was 2.5 cm or more in any single dimension. Human melanoma cell lines with fluorescent BRN2 reporter were further transduced with pHIV-Luc-ZsGreen (Addgene 39196) and selected with neomycin (800 μg/mL) for 3 weeks. GFP high expressing cells were isolated by FACS and expanded. mCherry reporter high and reporter low cells were then FACS isolated. After brief expansion in culture, 1–2 million cells from each group were injected into the flank of adult male or female NOD *scid* gamma mice at a site distant from the lungs. Tumor growth was monitored twice weekly with a digital caliper. For flow studies, tumors were excised, dissociated and de-moused,^77^ then analyzed with a BD Fortessa. For luciferase studies, mice were imaged in a PerkinElmer IVIS Spectrum Imaging System for luminescence every 2 weeks, 15 min after subcutaneous injection of 100 μL (150 mg Luciferin/kg body weight) of D-luciferin (Goldbio LUCK-1G). After the final week, mice were immediately sacrificed and organs were harvested and imaged. All animal studies were conducted by the Huntsman Cancer Institute Preclinical Research Resource or UCSF Preclinical Therapeutics Core, of which the technicians were blinded to the cell types and hypotheses.

#### Single cell RNA sequencing library preparation

##### BD rhapsody mRNA whole transcriptome qnalysis library preparation

Single-cell suspensions were processed using the BD Rhapsody Single-Cell Analysis System according to the manufacturer’s instructions (*BD Rhapsody System mRNA Whole Transcriptome Analysis Library Preparation Protocol*, BD Biosciences). 1 × 10^3^ - 2 × 10^4^ viable cells were loaded into BD Rhapsody cartridges containing cell capture beads functionalized with oligonucleotides carrying both unique molecular identifier and cell-specific barcodes. After single-cell capture, lysis was performed within the cartridge, and bead-bound mRNA transcripts were reverse-transcribed on-bead to generate barcoded cDNA molecules. Following Exonuclease I treatment to remove unextended primers, beads were transferred into the BD Rhapsody Whole Transcriptome Amplification (WTA) workflow for random priming and extension (RPE) using the BD Rhapsody WTA Amplification Kit (Cat. No. 633801).

During the RPE step, double-stranded barcoded cDNA was synthesized and amplified through sequential temperature-controlled incubations to denature, anneal, and extend fragments. Amplified cDNA was magnetically separated and purified using a single-sided AMPure XP bead cleanup (Beckman Coulter) to remove low molecular weight products. The purified amplicons were used as input for index PCR, which incorporated Illumina-compatible adapter sequences and dual sample indices using BD-supplied primers. The number of PCR cycles was adjusted according to cell input (typically 12–14 cycles for 10^3^-10^4^ cells). A dual-sided AMPure XP cleanup was then performed to enrich fragments in the 250–1,000 bp size range and eliminate adapter dimers or nonspecific products.

##### ScaleBio single cell RNA sequencing Kit v1.1

Cells were processed using the ScaleBio Single-Cell RNA Sequencing Kit (v1.1; ScaleBio Inc.), which employs a split-pool combinatorial indexing strategy to barcode cells across multiple rounds of processing without microfluidic instrumentation. This workflow uses a series of plate-based reactions in which fixed or live single cells are distributed across wells containing indexed reagents for successive rounds of barcoding. Each cell receives a unique barcode combination during three steps: reverse-transcription barcoding, ligation barcoding, and tagmentation/indexing PCR, yielding >3.5 million unique barcode permutations per experiment. Protocol supports processing up to ~125,000 cells per run and allows for multiplexing of up to 96 samples in a single experiment.

After reverse transcription, cDNA was purified and ligated to secondary barcodes using ScaleBio’s proprietary reagents, followed by cleanup with magnetic beads between each step. Final amplification was performed using limited-cycle PCR to incorporate Illumina-compatible adapters and indices, producing sequencing-ready libraries. Cleanup and size selection were carried out using AMPure XP magnetic beads to remove small fragments and primer dimers. Library yield and size distribution were evaluated by Qubit dsDNA quantification and Agilent Bioanalyzer analysis.

#### Single cell RNA sequencing

scRNASeq libraries were prepared from freshly disassociated cell cultures according to the BD Rhapsody System mRNA Whole Transcriptome Analysis or ScaleBio Single Cell RNA Sequencing Kit according to the above protocols (selected parameters: 11 cycles for sample tag PCR1 and 13 cycles for RPE PCR) and sequenced on an S4 Flow Cell of a NovaSeq6000.

Raw data were processed using the BD Rhapsody WTA Analysis pipeline which generates BAM files and single count matrices from raw FASTQ files. The BD Rhapsody output files (RSEC_MolsPerCell and Sample_Tag_Calls) were loaded into the Seurat 4.2.0 package in R.^78^ Cells from 624Mel mCherry low and high were normalized using the sctransform v2 method^79^ and clustered using 15 dimensions and a 2.0 resolution with UMAP. Differentially expressed genes were identified using the default Wilcoxon Rank-Sum test in Seurat. Module scores were added using ‘AddModuleScore’ in Seurat to identity gene sets with higher or lower expression than a random set of 100 genes.^35^ Cell cycle scores were calculated using ‘CellCycleScoring’ in Seurat with the 2019 update of cell cycle genes. High-coverage scRNAseq was conducted with the ScaleBio Single Cell RNA Sequencing Kit v1.1. Fastq files were created using BCL Convert with the ScaleRNA_3L_samplesheet_v1.1.csv file on Github (https://github.com/ScaleBio/ScaleRna/blob/master/docs/examples/fastq-generation/ScaleRNA_3L_v1.1/ScaleRNA_3L_samplesheet_v1.1.csv). Fastq files were aligned to the ScaleBio GRCh38 reference (http://scale.pub.s3.amazonaws.com/genomes/rna/grch38.tgz) using ScaleRna 1.6.3 (https://github.com/ScaleBio/ScaleRna) to create QC reports and filtered gene barcode matrices. Filtered gene barcode matrices were clustered in Seurat and gene signature scores were calculated with AddModuleScore against a background set of genes. Pearson correlation coefficients (*r*) were computed between feature scaled program scores using SciPy’s pearsonr test, which also provides a *p*-value testing against the null hypothesis of zero correlation. Additionally, to assess the dediff–Myc relationship at controlled diff levels, cells were binned by DIFF expression and calculated within-bin correlations.

The cell annotations and count files from Jerby-Arnon et al.^37^ and Belote et al.^36^ were downloaded from GSE115978 and GSE151091 at NCBI GEO and respectively combined into Seurat objects. For the Belote dataset, only adult cutaneous skin specimens were used. Of these, samples with fewer than 26 cells were excluded from the analysis (*n* = 2). The remaining datasets were split by sample identity for integration using Seurat’s SCTransform (SCT) workflow. Integration features were selected using 3,000 highly variable genes, and the datasets were prepared for SCT-based integration. Integration anchors were identified, and the datasets were merged using a weighted nearest-neighbor approach (*k.weight* = 75). Dimensionality reduction was performed using principal component analysis (PCA), followed by Uniform Manifold Approximation and Projection (UMAP) for visualization. Cell-cell relationships were inferred using nearest-neighbor graphs, and clustering was performed based on the integrated transcriptomic profiles. For all datasets, the counts were normalized using the global-scaling normalization and module scores were added using AddModuleScore. Classification correlation was conducted using the WIMMS portal.^58^ Pseudotime orderings were obtained by calculating centroids, constructing a minimum spanning tree and plotting pseudotime values starting at specific clusters following published code.^80^ Velocity vectors were added to the Seurat object by loading a loom file with spliced and unspliced counts, calculating cell-cell distances and estimating velocities using the velocyto.R package, followed by imputation.^65^

#### SphereDrop outgrowth assay

After sorting, clonal populations of BRN2 high and BRN2 low cells were prepared as spheroids in a nucleon Sphera U bottom plate (Thermo Scientific, Cat. No 174925) at 50 cells/well. After 4 days, spheroids were transferred to a 48 well plate coated with 0.01 mg/mL fibronectin (Corning 354008). Coating was prepared in cold sterile PBS and applied for 1 h at 37°C in a humidified incubator, washed 2× with PBS, rinsed with deionized water, dried, and stored at 4°C for up to 1 week until ready to plate cells. To transfer the spheres, a 20 μL drop of media was placed in the center of a well, the sphere, resuspended in 10–20 μL of media was added to the droplet. Spheres were allowed to settle and adhere to the plate for 2 h at 37°C in a humidified incubator. Immediately prior to imaging, media was very gently added to the well taking care not to disturb the sphere. Spheres were imaged on a Livecyte quantitative phase microscope (Phasefocus) for 24 h with an image acquired every 30 min. To count the cells that left the sphere, images were analyzed in the cell analysis toolbox (Phasefocus) by masking out the sphere and dilating the mask so the boundary extends past the spheroid equivalent to 1 cell diameter. Cells outside of the masked region were segmented and counted at each timepoint. For area analysis, phase images were segmented and analyzed in FIJI^72^ and further calculations were performed in MATLAB. Briefly, images were smoothed, thresholded by Huang2, binarized, expanded, filled holes, eroded, and despeckled. Remaining objects >200 μm^2^ were analyzed and the final calculations for the change over time were performed in MATLAB.

### QUANTIFICATION AND STATISTICAL ANALYSIS

#### Statistical analyses

Statistical analyses were performed using GraphPad Prism 8 software (GraphPad Software Inc.) unless otherwise noted. Data are presented as mean ± standard deviation unless otherwise noted in figure legends. The value of “n” and what it represents (e.g., number of biological replicates, experiments, individual cells, mice, etc.) are reported in each figure legend. Statistical tests were chosen based on data distributions and sample variance types: Unpaired t-tests were used for Gaussian distributions, Mann-Whitney tests for non-Gaussian distributions or for samples of unequal variance and pairwise Chi-Square tests for comparing proportions. Exact *p* values were calculated as reported by Prism 8 (Graphpad) and reported as: *p < 0.05; **p < 0.01; ***p < 0.001; ****p < 0.0001; ns, no significant difference.

## Supplementary Material

1

2

3

4

5

6

7

SUPPLEMENTAL INFORMATION

Supplemental information can be found online at https://doi.org/10.1016/j.celrep.2025.116675.

## Figures and Tables

**KEY RESOURCES TABLE:** 

REAGENT or RESOURCE	SOURCE	IDENTIFIER

Antibodies		

POU3F2 (BRN2) (1:1000 dilution 5% BSA)	Cell Signaling	Cat#: 12137S; RRID: AB_2797827
AXL (1:1000 dilution 5% BSA)	Cell Signaling	Cat#: 8661; RRID: AB_11217435
MITF (1:1000 dilution 5% BSA)	Cell Signaling	Cat#: 12590; RRID: AB_2616024
c-MYC (1:300)	Cell Signaling	Cat#: 9402; RRID: AB_2151827
phosphorylated c-MYC (1:300)	Cell Signaling	Cat#: 13748; RRID: AB_2687518
Vinculin (1:1000 dilution 5% BSA)	Cell Signaling	Cat#: 4650; RRID: AB_10559207
GAPDH (1:1000 dilution 5% BSA)	Cell Signaling	Cat#: 2118; RRID: AB_561053
HSP90 (1:1000 dilution 5% BSA)	Abcam	Cat# 13495; RRID: AB_1269122
Goat anti-mouse secondary antibody-HRP (1:10,000 5% milk)	Invitrogen	Cat#: G-21040
Goat anti-rabbit secondary antibody-HRP (1:10,000 5% milk)	Invitrogen	Cat#: G-21234
Goat anti-rabbit Alexa Fluor Plus 488 (1:10,000 5% milk)	Invitrogen	Cat#: A32731

Chemicals, peptides, and recombinant proteins		

Triton X-100	Sigma Aldrich	Cat#: T8787
Polybrene	Sigma Aldrich	Cat#: TR-1003
Neomycin (G418)	Sigma Aldrich	Cat#: A1720
Trizol	Ambion	Cat#: 15596026
chloroform	Sigma Aldrich	Cat#: 472476
isopropanol	Acros	Cat#: 67-63-0
D-luciferin	Goldbio	Cat#: LUCK-1G
RPMI1640	UCSF Core Facility	Cat#: CCFAE001
Cell-Tak Cell and Tissue Adhesive	Corning	Cat#: CB-40240
DMEM	Corning	Cat#: 10-013-CV
Dextran sulfate	Sigma Aldrich	Cat#: 42867
Formamide	Thermo Fisher	Cat#: AM9342
Formaldehyde 20%	Tousimis	Cat#: 1008A
DAPI	Thermo Fisher	Cat#: D1306
Trypsin EDTA	Mediatech	Cat#: MT 25-052-C1
10× PBS, Rnase free	Life Technologies	Cat#: AM9624
20× SSC	Thermo Fisher	Cat#: 15557044
Glucose	Sigma Aldrich	Cat#: G7021
Tris(1M) ph8, Rnase free	Life Technologies	Cat#: AM9855G
Glucose Oxidase	Sigma Aldrich	Cat#: G0543-10K
6-Hydroxy-2,5,7,8-tetramethylchromane-2-carboxylic acid (Trolox)	Sigma Aldrich	Cat#: 238813
Catalase	Sigma Aldrich	Cat#: C3156-50
Donkey Serum	Jackson Immuno Research Labs	Cat#: 017-000-121
BSA	Sigma Aldrich	Cat#: A9647

Critical commercial assays		

Pierce^™^ BCA protein assay kit	Thermo Fisher	Cat#: 23225
Sensifast cDNA synthesis kit	Bioline	Cat#: Bio-65053
Sensifast SYBR non-Rox kit	Bioline	Cat#: Bio-98005

Deposited data		

GSE150582	NCBI Gene Expression Omnibus	https://www.ncbi.nlm.nih.gov/geo/query/acc.cgi
GSE230574	NCBI Gene Expression Omnibus	https://www.ncbi.nlm.nih.gov/geo/query/acc.cgi

Experimental models: Cell lines		

624Mel	Gift from Dr. Boris Bastian	CVCL_8054
SK-MEL28	Gift from Dr. Boris Bastian	CVCL_0526
WM793	Gift from Dr. Meenhard Herlyn	CVCL_8787

Experimental models: Organisms/strains		

Immunodefecient NOD scid gamma mice	UCSF and HCI preclinical cores	N/A

Oligonucleotides		

See Table S4 for oligonucleotides used in this study		N/A

Software and algorithms		

Graphpad Prism 6.0	GraphPad Software, Inc.	https://www.graphpad.com/scientific-software/prism/
FlowJo V10	FlowJo LLC.	http://www.flowjo.com/
DHC analysis software	DHC.	http://phiab.com
Photoshop 2019	Adobe	www.adobe.com
Illustrator 2019	Adobe	www.adobe.com
Stellaris^®^ Probe Designer version 4.2	LGC Biosearch Technologies	http://www.singlemoleculefish.com/
Fiji	Schindelin et al.^72^	https://imagej.net/Fiji/Downloads
HStudio	Phase Holographic Imaging PHI AB	https://phiab.com/holomonitor/cell-imaging-software/
Loon53	University of Utah	https://loon.sci.utah.edu/

## Highlights

- Method for prospective isolation of distinct melanoma transcriptional states
- Discovery of BRN2:MYC axis that interconverts through an intermediate MITF state
- BRN2 state is invasive and drug resistant, whereas MYC state is tumorigenic
- Healthy melanocytes share some states with melanoma but are less plastic
