# Supplementary material for: A BRN2:MYC transcriptional axis regulates interconversion between therapy-resistant and tumorigenic phenotypes in melanoma
Source: Cell Rep. Author manuscript; Available in PMC 2026 Jan 26. (PMC12834598; doi:10.1016/j.celrep.2025.116675)
Supplement: 1 [file NIHMS2132756-supplement-1.pdf]

Cell Reports, Volume *44*

Supplemental information

A BRN2:MYC transcriptional axis regulates interconversion between therapy-resistant and tumorigenic phenotypes in melanoma

Yuntian Zhang, Marcus A. Urquijo, Rebecca G. Zitnay, Kayla Marks, Rachel L. Belote, Maike M.K. Hansen, Montana Ferita, Hannah M. Neuendorf, Tong Liu, Eric A. Smith, Elnaz Mirzaei Mehrabad, Miroslav Hejna, Tarek E. Moustafa, Devin Lange, Min Hu, Fatemeh Vand-Rajabpour, Anne Done, Carly A. Becker, Matthew Lieberman, Matthew Chang, Brian K. Lohman, Chris J. Stubben, Melissa Q. Reeves, Xiaoyang Zhang, Leor S. Weinberger, Matthew W. VanBrocklin, Dekker C. Deacon, Douglas Grossman, Benjamin T. Spike, Alexander Lex, Glen M. Boyle, Rajan Kulkarni, Thomas A. Zangle, and Robert L. Judson-Torres

Supplemental Figure 1: Supporting data related to Figure 1. A) Validation of BRN2 IF fluorescence intensity quantification. Data represent the distribution of integrated IF intensity (arbitrary fluorescence units) from individual nuclei identified with DAPI. Shown are 624-mel cells either transduced with shBRN2, not treated, transduced with a BRN2 over expression (OE) construct, and secondary antibody only controls. Box and whisker plot with middle bar plotted at median, box edges at interquartile range, and whiskers set to min and max values. B) Quantification of the intensity of BRN2 IF (arbitrary fluorescence units of individual cells) from independent 624-mel clonal populations measured after 2 weeks (before arrow) and then 2 months (after arrow) expansion. Dotted line is for reference. C) Flow analysis of clonally expanded then serially cultured SK-MEL-28 and WM793 cells with the BRN2 reporter. D) Average and standard deviation of ∆Ct between probes for BRN2 and RPL37A transcript RT-qPCR probe sets. Mean mRNA expression plotted with standard deviation, N = 5 (624MEL and SK-MEL-28) 6 (WM793), number of independent RNA preparations, P, two-tailed t test P value.

Supplemental Figure 2: Supporting data related to Figure 2. A) Classification of indicated cell lines into mCherry high ($High^{BRN2}$) and mCherry low (Low^{BRN2}) populations using a linear discriminant analysis (LDA) of morphologic features. Population distribution, classification accuracy and p value from two-tailed t test (P) are shown. B) Instantaneous cell velocity over time of $High^{BRN2}$ and Low^{BRN2} cells assessed with quantitative phase imaging (QPI). The population mean and standard deviation of three independent experiments are shown. A two-tailed t test revealed no difference (N.D.) between the populations. C) Quantification of spheroid outgrowth assays as depicted in Fig. 2C. Area of the sphere outgrowth is measured over time. Mean area plotted with standard deviation, P-value determined by two-tailed t test. N = 4 (all cell lines) D) Growth rate (% volume increase / day) of all tumors that formed, stratified by injected cells. Box and whisker plot with center line representing median, boxes as interquartile ranges, and whiskers minimum and maximum values. P-value determined by two-tailed t test. E) Day of first palpable tumor detection, stratified by sex. Box and whisker plot with center line representing median, boxes as interquartile ranges, and whiskers as minimum and maximum values. P-value determined by two-tailed t test. F) Growth rate of all tumors that formed, stratified by sex. Box and whisker plot with center line representing median, boxes as interquartile ranges, and whiskers minimum and maximum values. P-value determined by two-tailed t test. G) The percent of mCherry high ($High^{BRN2}$) and mCherry low (Low^{BRN2}) cells detected by flow of disassociated primary or distant metastatic tumors. Mean growth rate plotted with standard deviation, P-value determined by two-tailed t test. N = 4. H) Schematic of luciferase assay. Clonal 624-mel cultures were either depleted (red) or enriched (gray) for Low^{BRN2} cells and implanted into NOD scid gamma mice. Luciferase imaging was conducted 1, 3, and 5 weeks after implantation, followed by sacrifice and organ imaging. I) Representative full mouse images of luminescence at indicated time points. J) Representative organ images (brain, liver, and lungs) of luminescence after week 5. K) Percent of mice with successful grafts after 5 weeks. P, two-tailed Fisher's exact test P value with 0.98 power. L) Average (line) and individual summations of luciferase intensity in brain, liver, and lungs. P-value determined by two-tailed t test. N = 5 Low^{BRN2} and 7 $High^{BRN2}$ M) Normalized mean single-cell mass over time of FACS-enriched $High^{BRN2}$ (left) and Low^{BRN2} (right) 624-mel cells exposed to indicated concentrations of vemurafenib. The asterisk indicates slopes significantly different from DMSO condition ($P < 0.0005$, simple linear regression).

Supplemental Figure 3: Supporting data related to Figure 3. A) Monitoring of cellular dry mass (relative) and cellular sphericity (0-1) over the course of one cell cycle. Horizontal axis ticks indicate 15-minute intervals. Horizon plots depict values as stacked layers: as values exceed thresholds, they wrap to the baseline and are represented in progressively darker shades, illustrating relative magnitude while preserving compactness. B) Tracking of cellular dry mass (relative) and cellular sphericity (0-1), and integrated mCherry expression level (relative) from parental cell to daughter cells. A characteristic pattern of gradual mass doubling (blue) followed by a rapid halving of mass coupled with a spike of sphericity (green) permits bookmarking of cycle cycles. C) Examples full cell cycles identified via the

reproducible pattern of cellular dry mass and cellular sphericity during the course of cell growth and division. mCherry expression does not present a similar cyclic pattern.

Supplemental Figure 4: Supporting data related to Figure 4.
A) Pseudotime values for clusters along the predicted transitions between termini, initiating from each of the three termini. B) Dendrogram depicting transcriptional signatures clustered by correlation coefficients. Clusters identified in Hu 2024 are indicated in blue (differentiated), red (MYC/mitosis) and green (dedifferentiated). Termini identified by RNA velocity analysis are indicated by black arrows. C,D) UMAPs overlaid with indicated signature enrichments from Simmons 2017, Tirosh 2016, Wouters 2020, and Rambow 2018. E) Western blot analysis of cMYC and phospho-cMYC in FACS-enriched $High^{BRN2}$ (H) and Low^{BRN2} (L) cells. F) Percentage of $High^{BRN2}$ or Low^{BRN2} cells in the DEDIFF terminus (green), the MYC terminus (red) or MITF high clusters (blue). F) Western blot analysis for MITF and AXL expression in FACS-enriched $High^{BRN2}$ (H) and Low^{BRN2} (L) cells.

Supplemental Figure 5: Supporting data for Figure 5. UMAP visualization of melanocytes from healthy adult skin identifies seven Seurat clusters (A), with overlays showing backscatter (BSC) data correlating with melanin content (B), pseudotime trajectories (C), and the distribution of melanocytes from 12 individual adult skin specimens across clusters (D).

Supplemental Figure 6: Supporting data related to Figure 5. A-B) Quantification of rate of change in relative integrated mCherry normalized to dry mass in either primary melanocytes (A) or melanoma cells (B). Dry mass and duration filters (see methods) were applied to 956 melanocyte and 1168 melanoma cell lineages, resulting in 56 melanocyte and 301 melanoma cells being analyzed. Slopes for normalized mCherry change were calculated across time (hours) with melanocytes: min= -19.9928, 1st quartile= -3.7615, median= -0.3551, mean=-0.5513, 3rd quartile = 1.6488, max= 17.0926 and melanomas: min= -430.90, 1st quartile= -27.40, median = 13.87, mean= 17.49, 3rd quartile= 50.96, max= 335.92, indicating melanoma cell lines are transitioning reporter states more readily than melanocytes. Twenty-four representative plots are shown. C) Scatterplot of rate of normalized mCherry change in melanocytes (NHM LP051) and melanoma (624-mel clone 1) over time (hours) showing all data points. D) Empirical Cumulative Distribution Function (ECDF) comparing normalized mCherry change across four NHM lines (LP051, 1-3, DP02, FS007, n = 2711) to six melanoma lines (624-mel clones 1, A1-3, B1-1, B3-2, and SK-MEL-28 clones A1 and A2, n = 1143). Two-sample Kolmogorov-Smirnov test $p<2.2E-16$. E) Comparison of the linear regression model (LM) and the linear mixed effects (LME) model for data in C.

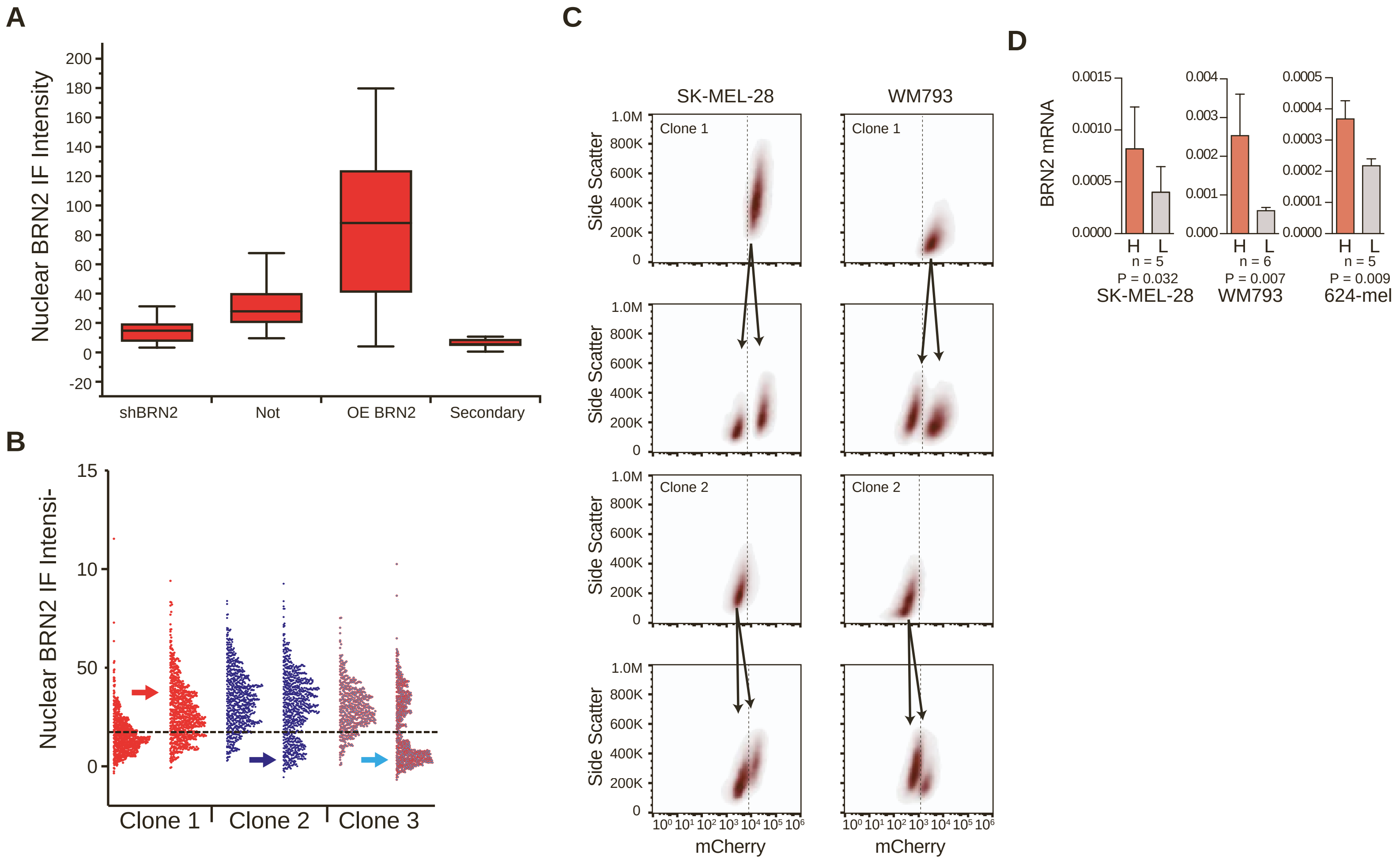

Supplemental Figure 1: Supporting data related to Figure 1

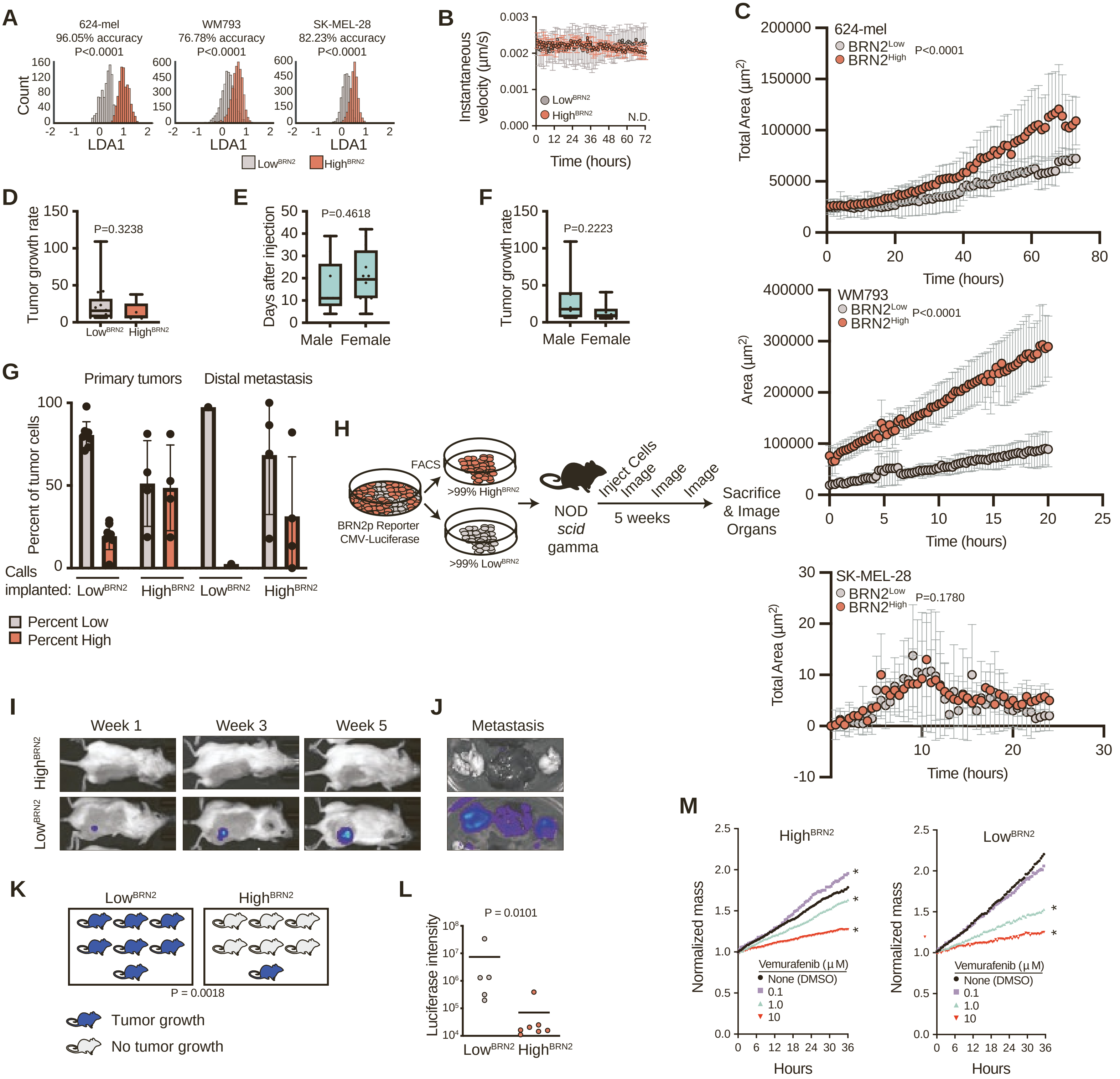

Supplemental Figure 2: Supporting data related to Figure 2

A

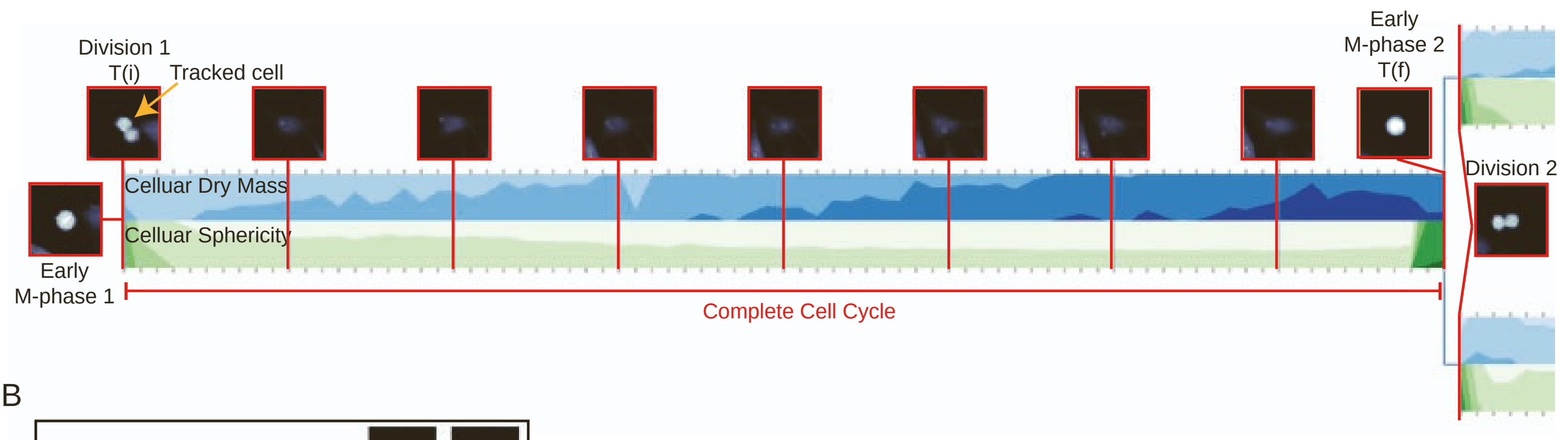

B

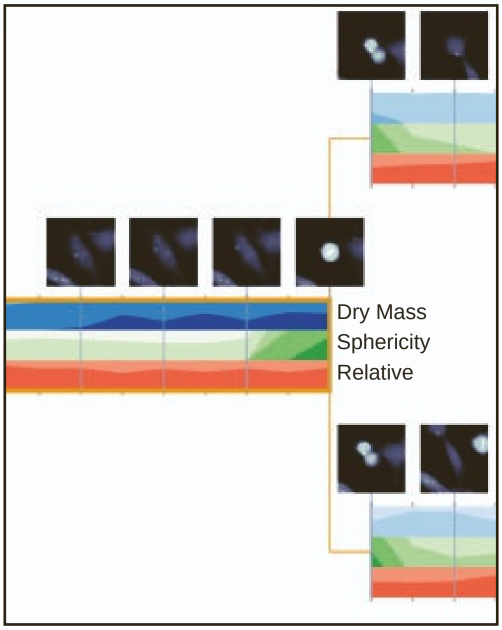

C

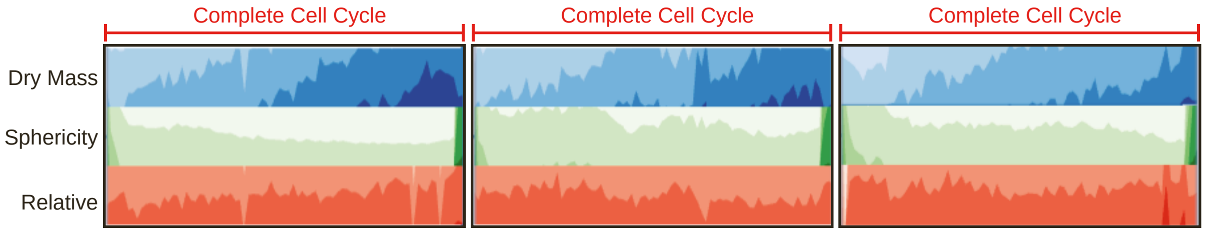

Supplemental Figure 3: Supporting data related to Figure 3

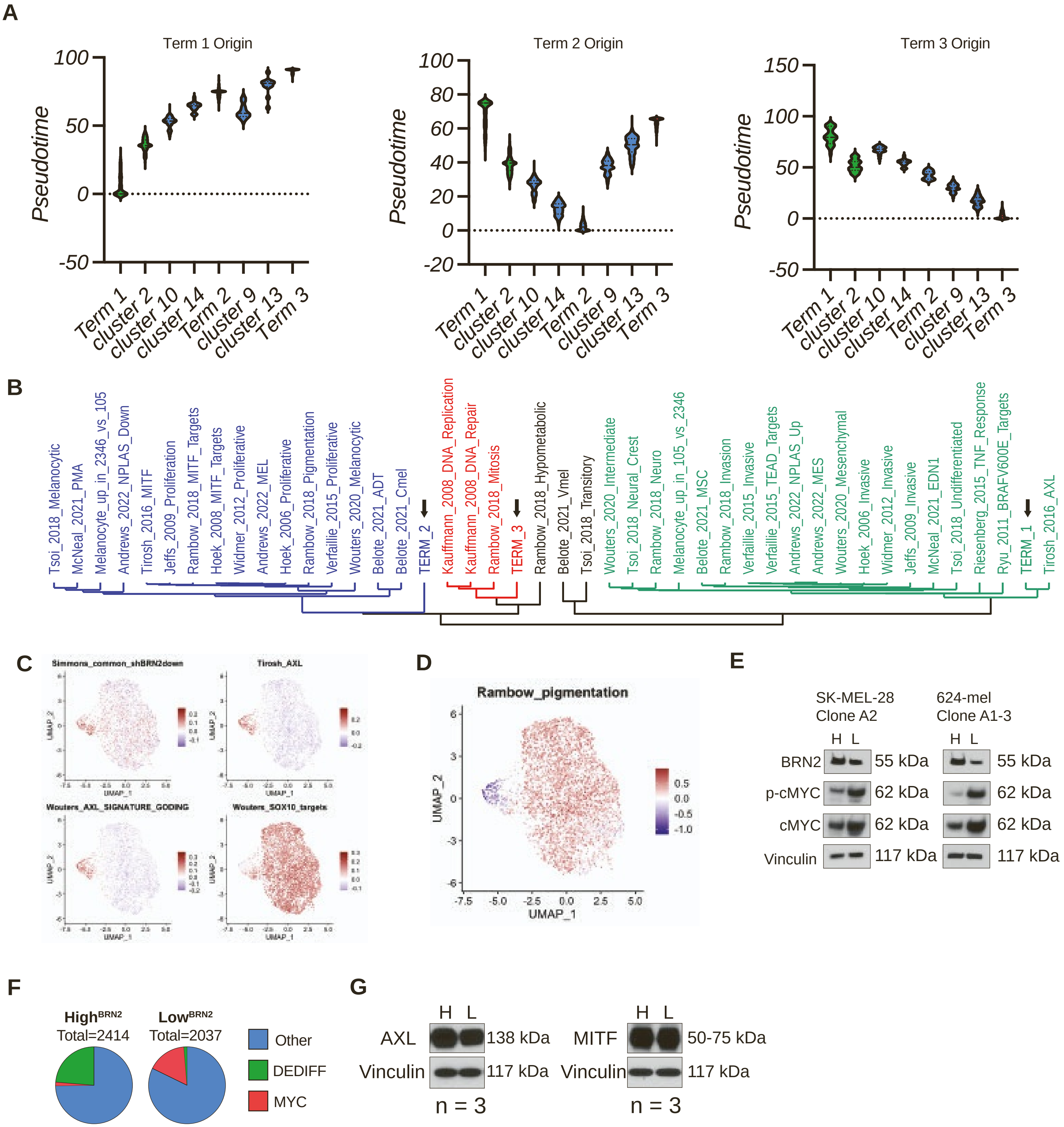

Supplemental Figure 4: Supporting data related to Figure 4

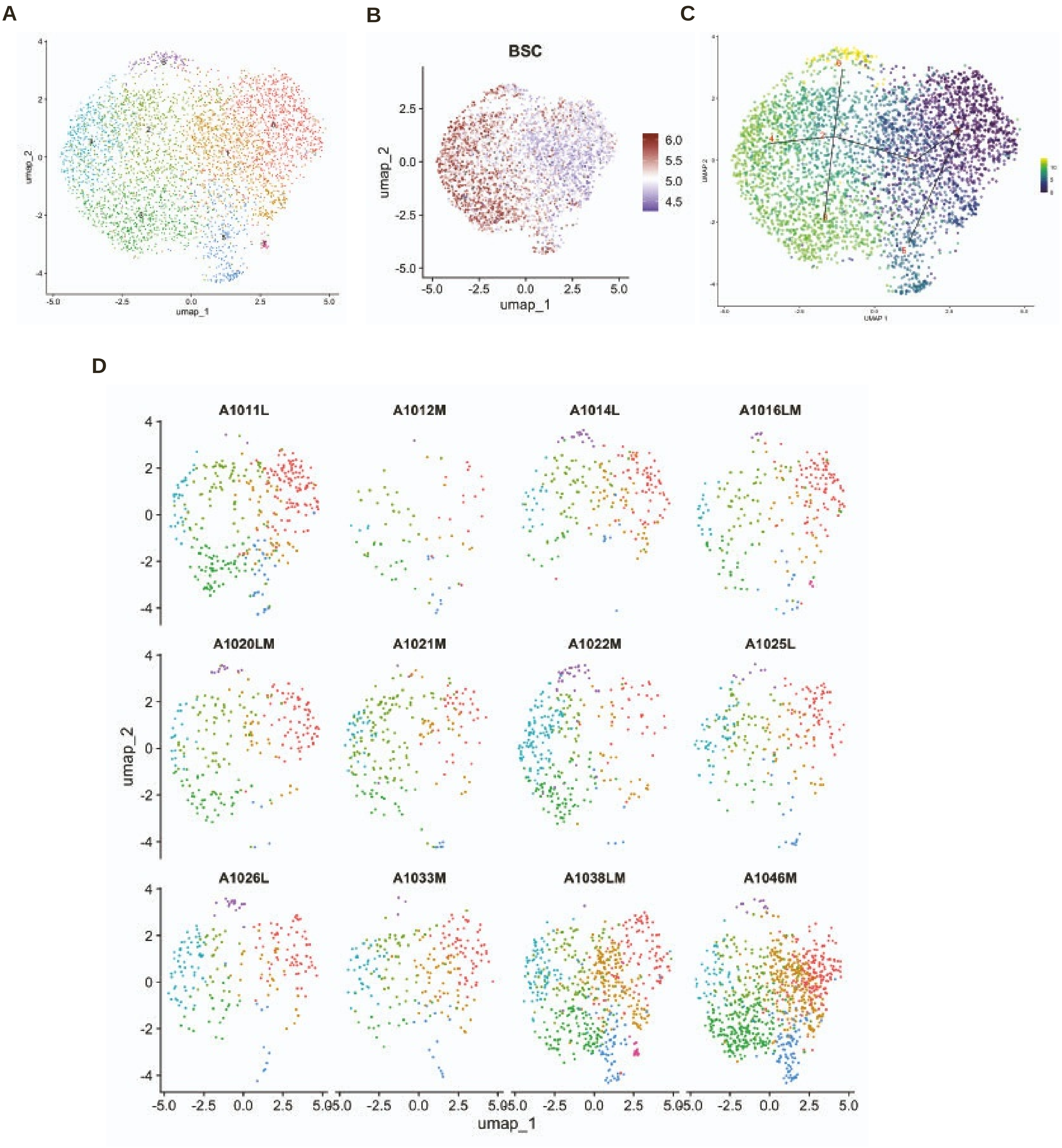

Supplemental Figure 5: Supporting data related to Figure 5

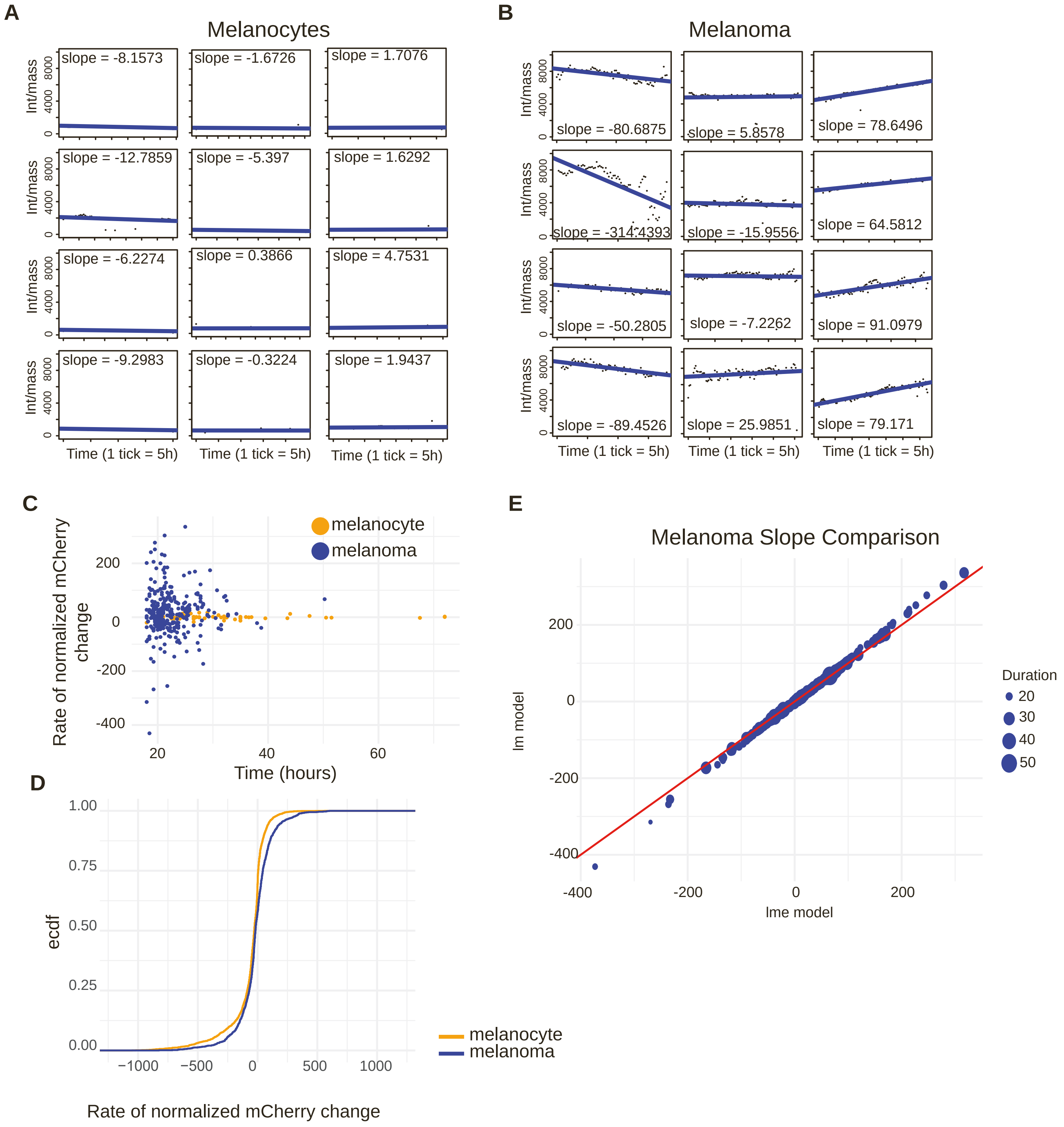

Supplemental Figure 6: Supporting data related to Figure 5
